# Progress to extinction: increased specialisation causes the demise of animal clades

**DOI:** 10.1038/srep30965

**Published:** 2016-08-10

**Authors:** P. Raia, F. Carotenuto, A. Mondanaro, S. Castiglione, F. Passaro, F. Saggese, M. Melchionna, C. Serio, L. Alessio, D. Silvestro, M. Fortelius

**Affiliations:** 1 Department of Earth Science, Environment and Resources, University of Naples Federico II, 80138 Napoli, Italy; 2 Department of Biological and Environmental Sciences, University of Gothenburg, 413 19 Gothenburg, Sweden; 3 Department of Geosciences and Geography, P.O. Box 64, FI-00014 University of Helsinki, Finland; 4 Centre for Ecological and Evolutionary Synthesis, Department of Biosciences, University of Oslo, P.O. Box 1066 Blindern, NO-0316 Oslo, Norway

## Abstract

Animal clades tend to follow a predictable path of waxing and waning during their existence, regardless of their total species richness or geographic coverage. Clades begin small and undifferentiated, then expand to a peak in diversity and range, only to shift into a rarely broken decline towards extinction. While this trajectory is now well documented and broadly recognised, the reasons underlying it remain obscure. In particular, it is unknown why clade extinction is universal and occurs with such surprising regularity. Current explanations for paleontological extinctions call on the growing costs of biological interactions, geological accidents, evolutionary traps, and mass extinctions. While these are effective causes of extinction, they mainly apply to species, not clades. Although mass extinctions is the undeniable cause for the demise of a sizeable number of major taxa, we show here that clades escaping them go extinct because of the widespread tendency of evolution to produce increasingly specialised, sympatric, and geographically restricted species over time.

Most animal clades follow a predictable path in geographic commonness and taxonomic diversity over time^1^,^2^. Clades usually start within a very restricted range^3^, and then expand and diversify to occupy large stretches of Earth. Almost immediately after this peak in success, they start declining in diversity and finally go extinct^1^,^2^,^4^,^5^,^6^ (although a few may survive with as little diversity as one genus like sphenodonts, coelacanths, and nautiloids). There is no consensus about the reasons underlying this path, and no general explanation has been advanced as to why clades have to die out following such a predictable path^7^. Studies of paleontological extinctions deal with the ever-increasing negative effect of biotic interactions^8^, geologic and climatic events^1^,^2^, ecological and evolutionary traps^9^, and of course major extinction crises^10^. While these factors certainly represent effective causes for extinction of species, their impact at the level of clades is unclear, although it has been demonstrated that competition may drive entire clades extinct^11^,^12^. With the exception of clades that fail during mass extinctions, we argue the explanation for the demise of most other clades lies in the tendency of natural selection to produce more and more specialised types within each lineage as time progresses^13^,^14^, a pattern we dubbed weak directionality (in contrast to old views of evolution as moved towards the “perfection of life”)^13^. Herein we define specialisation as a process of adaptation to a specific habitat via the acquisition of narrow ecological niches^15^, and infer the degree of specialization by the dynamics of species range size and its mutual overlap among species within clades. While specialization confers obvious advantages in terms of competitive abilities where and when species do live^16^, it also increases extinction risk^17^,^18^,^19^,^20^,^21^, via reduction in range size^9^,^10^,^22^, and by depressing cladogenesis^23^,^24^. According to this “weak directionality” model^13^, clade geographic range and taxonomic diversification is best viewed as proceeding according to two distinct phases (Fig.1): during the *early phase* species richness increases, and the clade expands geographically. The intensity of competition and the extinction rate are low on average. But as the clade diversifies its total occupied range fills the available ecological space, thus depressing geographical expansion and greatly strengthening the effects of competition^7^,^8^,^24^,^25^. During this stage, specialisation might further foster taxonomic diversity, by promoting species coexistence, hence sympatry^26^,^27^,^28^. Yet, it also drives the clade into a *late phase* when its negative effects of survival and diversification will eventually take their toll on clade survival.

The most direct expectation of weak directionality is that clades should become rich in specialised species as time passes. Hence, under this theory, we predict that most species in the *late phase* of clade evolution should have traits typical of specialists, such as small range size and high degree of sympatry^17^,^18^,^29^,^30^. Given high degree of sympatry and reduced range size are presumed to depress diversification, according to this model there should be some point in time where both the regime of range size evolution and the diversification process shift (Fig. 1). We tested this hypothesis by locating statistically significant shift points in the total range size, degree of sympatry, and net diversification rate curves. We then tested four hypotheses consistent with the *early/late* phases scenario to assess whether 1) our data support the existence and temporal coincidence of total range size, degree of sympatry, and diversification shifts, 2) the degree of sympatry increases and the net diversification rate decreases after the shifts, and 3) the average species range size decreases after the shifts (see Fig. 1) and 4) the degree of sympatry is negatively correlated to speciation rate and positively correlated to extinction rate, which would indicate the link between specialisation and the decrease in diversification.

To test these predictions we collected from the Paleobiology Database (https://paleobiodb.org/#/ on 2/10/2016) the fossil occurrence data on 21 extinct animal clades belonging to five different phyla (Cnidaria, Mollusca, Brachiopoda, Arthropoda, and Bryozoa, see supplementary information). The data included 14,430 species and 84,454 fossil occurrences, spanning around 480 million years from the early Cambrian trilobites and brachiopods, to late Cretaceous ammonites.

For all of the analysed clades, we first computed the range size of each species per time bin, and the range size of the entire clade per time bin, which represents the union of individual species ranges (Fig. 2). We then summed individual species ranges within each time bin and then over consecutive time bins, to produce a “total range curve”. The use of cumulative range values, rather than time bin data, is appropriate as it smooth off unequal sampling and allows calculating effectively changes in the regime of geographical evolution of clade (see below). The (slope of) total range curve plotted versus time indicates the velocity of range size accumulation at the level of clade. It is equivalent to the average size of species ranges times species richness cumulated over all time bins. The total range curve is best fitted by either sigmoid, or generalized logistic curves, while the linear model is rejected for all the examined clades. This indicates that the increase in total range size slows down towards the recent, according to saturation dynamic (Table 1). To quantify the degree of sympatry (Figs 1B and 2), we started by summing the geographic range of the entire clade over consecutive time bins. This “clade range curve” is different from the total range curve in that it depends on how much individual species ranges do overlap (for instance, if two species have range = 1 km^2^ and do perfectly overlap the total range curve will be 1 + 1 = 2 km^2^, while the clade range curve will be 1 km^2^, Fig. 2). Then, we computed the area between total and clade range curves per unit time, under the specific hypothesis that the area difference between the two curves should be larger after the shift point, thereby indicating a higher degree of sympatry since (Fig. 1B). Eventually, we tested how often the difference between the two curves tends to increase after the shift points across clades, in keeping with our hypothesis 2, by means of the binomial distribution.

The net diversification rate was computed starting from the fossil record as the expected number of speciation/extinction events per lineage per Myr. Finally, we computed a second measure of the degree of sympatry at the level of bin. For each such bin, we took the ratio between the total range size (summed algebraically over all species in the bin) and the clade range size in the focal interval. This ratio represents the degree of overlap among individual species ranges. We assessed whether changes in the degree of sympatry throughout a clade’s history significantly correlates with temporal variation in speciation and extinction rates. To this aim, we fitted birth-death models in which speciation and extinction rates respond to changes in sympatry by means of an exponential correlation with parameters γ_λ_ and γ_μ_, respectively^11^.

Shift points in degree of sympatry among species, total range size and net diversification rate are statistically closer in time to each other than expected by chance in 20 out of 30 cases, and for 16 out of 21 clades (Table 2). Both figures are statistically different from chance according to the binomial distribution (Table 3), indicating that the existence and temporal coincidence of shift points are robust. We then took the average ages of the three shifts to get a single shift point, and tested hypotheses 2 and 3. In keeping with our predictions (hypothesis 2) the degree of sympatry increases after 28 out of 30 shift points. Even after excluding the earliest third of clade evolution (when a high degree of sympatry is expected because in a diversifying clade species tend to place close to each other on Earth’s surface^5^) we recover the same pattern. The increase is temporally coincident with shift points (Table 3).

Then, we tested the prediction that species average range size decreases after the shift points (hypothesis 3), by dividing the total cumulative range curve by the number of species present in each time bin. This allows testing the evolution of species average range size over time (Fig. 1A,C). After the shift points this curve has a slope significantly different from zero fifteen times, 12 of them being negative (i.e. the species average range size decreases towards the recent, Table 3), and in eight different clades. This is consistent with the idea that species after the shifts tend to be small-ranged and therefore specialists. Yet, after nearly one half of the averaged shifts (16/31, 51.6%) there was no significant pattern in average range size.

Net diversification rates decrease after the shift points in 28 out of 30 cases and for all of the clades, thus supporting our predictions.

Finally, as expected the extinction rates are positively correlated with the degree of sympatry in 12 clades out of 21, and the speciation rate is negatively correlated to sympatry in 13 clades (Table 4). Overall, 16 clades out of 21 show either decreased speciation or increased extinction as the degree of sympatry increases, in keeping with our prediction that sympatry (as a consequence of specialisation) depresses diversification to drive the clade extinct.

Overall, our results indicate that the distinction between an early and a late phase of clade evolution is useful, that the net diversification rate decreases consistently during the late phase, and that mainly specialist species, having high degree of range overlap to each other (sympatry), make up the majority of clade biodiversity after the shift points.

We repeated all of the analyses excluding species with less than 10 total occurrences, in order to rule out the possibility that what we perceive as rarity, is in fact lack of preservation. The results are available as supplementary information. On such a reduced dataset, we located 22 shift points for seventeen clades. The shift points are statistically significant in 19 out of 22 cases (86.4%), and net diversification rate is always lower after than before the shifts. Yet, the degree of sympatry after the shift is higher than before only 9 times, and the average range size is significant and negative just one time. Taken at face value, these latter results are not supportive of weak directionality. Yet, it must be noted that by excluding rare (or otherwise poorly sampled) species from the dataset, we effectively removed those species whose effect on clade range size evolution we were seeking to test.

## Specialisation as the force promoting both the progress and death of clades

Weak directionality theory derives from the idea that natural selection incessantly fine-tunes species to their environment. Specialisation is an almost naïve consequence of such tinkering. Its payoff is evident in the short run^13^,^14^,^16^ but over long periods of time, it acts as an evolutionary trap^9^. While this has long being noted when dealing with the fate of individual species, the impact of such a trap on the history of clades is massive. We show that the degree of geographic range overlap among species increases as clades grow older (and most specifically after the shiftpoints). In modern ecosystems, it was shown that niche specialisation promotes sympatry^17^,^18^,^30^. Hence, this result points to a non-random pattern of the incidence of specialized species over time.

We proved a direct link between sympatry and diversification, which taken together with the notion that sympatry tends to grow over time during the history of clades, is further, crucial evidence, that specialisation undermines clade survival in the long run. We expected sympatry to impact negatively on speciation rate, and positively on extinction rate, but only during the late phase of clade evolution (i.e. after the shiftpoints). In this regard, it is especially interesting to note that we found sympatry to correlate negatively to diversification rate 12 times, and positively only 3 times. As per speciation rate, we retrieved 13 negative correlations to the degree of sympatry, and only 4 positive correlations. Overall, this suggest that sympatry alone could be enough to explain the common observation that origination rate decrease over time for purely biological reason, even when diversity is low^4^,^31^,^32^. The slow-down in geographic expansion that clades experience over time suggests that this mechanism is especially important when the available space for allopatric speciation becomes limited^33^, which our data suggest should happen after the shift points. Obviously, the net diversification rate decreases towards the present in all of the clades we analysed. Yet, what is important is that we found this decline is temporally coincident with the increased level of sympatry. Patterns of decreased diversification towards the present have also been frequently inferred from phylogenies^34^,^35^,^36^ and explained in that context as the product of limited opportunities for speciation as clade history progresses towards the present^37^,^38^. However, our data suggest that increased habitat specialisation, hence higher sympatry are fundamental aspects of such trend in diversification.

Our model is inherently simple and based on ideas that have roamed through the scientific literature for decades. The concept of weak directionality, for example, is directly based on Edward Cope’s law of the unspecialised^13^ (i.e. the idea that clade start with small generalist species to end up with large-bodied specialized types). We believe that the main obstacles to their further development until now have been the difficulties of estimating changes in clade range size from fossil data, the lack of adequate fossil databases, and a certain reluctance to use any concept of progress (i.e. strong directionality) in modern evolutionary thinking^39^.

Sewall Wright’s and George G. Simpson’s concepts of the evolutionary landscape^7^,^40^ are consistent with weak directionality theory. The landscape metaphor emphasizes the trend towards higher-fitness morphologies and genotypes as drivers of weak directionality. Here we have shifted the focus from how species evolve in coexistence by specialising on particular resources/environmental conditions as species richness builds up to the long-term, macroevolutionary effect of this process. Specialisation has often being envisaged as an evolutionary trap^9^ but its potential contribution to clade extinction has been largely neglected since Cope first identified the link between specialisation and clade demise in his *law of the unspecialised*.

Liow^41^ found that long-lived crinoid taxa are less specialised in morphology than more derived types (but see ref. 42). This bolsters the idea that specialisation increases extinction risk, and would support our interpretation here if such long-lived taxa are also stratigraphically older. Evidence for such a pattern comes from a study of ours on placental mammals^14^ where we demonstrated that specialised types are geologically younger than their relatives. Statistical modelling of the evolution of specialisation further support the idea that natural selection on local adaptation and habitat choice always leads to specialists, implying the latter appear later, on average, than generalists^43^.

We will not push our model so far as to say that all of the clades have to follow the path it predicts. According to the weak directionality theory, after the shift point the geographic evolution of clades proceeds towards a condition where a few, highly-sympatric, species coexist within a relatively small range. With the exception of the prediction for reduced average range size after the (mean) shiftpoint, we found complete adherence to our predictions for 15 out of 21 clades (binomial distribution, p = 0.026, Table 3). This is especially robust considering that a number of clades went extinct during a mass extinction, which means their natural course towards extinction was abruptly interrupted by something unrelated to range size dynamics within clade of any other biological attribute^10^, and the effect that specialisation had upon them. Although this dynamic is true for most of the clades we analysed - rhynconelliform brachiopods (Strophomenida, Spiriferinida, Orthotedida, and Productida), tabulate corals (Favositida), stenolaemate bryozoans (Fenestrida, Cystoporida, Trepostomida, Rhabdomesida), pteriacean bivalves (Pterineidae), proetid trilobites (Proetida), murchinsonid (Lophospiridae), eogastropod (Euomphalidae) and bellerophontid gastropods (Bellerophontida), and ammonoid ammonites (Desmoceratida) - there are also clades whose total range size keeps growing after the shift points, since the number of species remains high then - Auloporida (tabulate corals), Rhabdomesida (stenolaemata bryozoans), Strophomenida and Orthotetida (rhynconellid brachiopods). Not surprisingly, these are all clades whose species richness was still high in the late phase of their evolution (see supplementary material for the geographic and net diversification rate paths of individual clades).

It must be emphasized that whereas the evidence for increased sympatry and reduced diversification after the shift points is very robust (pertaining to more than 90% of the shifts), the comparable figure for the expected average range size decrease is extremely weak, as the results conform to the expectations only in 12 out of 30 shift points (40% of the cases). Indeed, the range size frequency distribution is usually right-skewed within clades^44^. This means that (relatively) large-ranged species are always expected to occur within a clade. In addition, after the shift point the decrease in species diversity implies that surviving species probably have the opportunity to occupy the range they left vacated. If the clade range size does not decrease in the latter bins, such dynamics would imply that average range size would not decrease in many cases. Thus, expectation of reduced average range size for species over time is too simplistic^45^.

Most of the clades mentioned so far conform to the predictions of the weak directionality model: they show high levels of sympatry, small average range size, and small total range size after the shift points. A manifold of taxa, though, show small total ranges, small average range and small degree of sympatry. These clades survived what according to the model should have been their final extinction moment because of species having disjunctive ranges. Thus, although rare, there are cases of clades whose species become rare overall in their former ranges, rather than becoming restricted to sympatry in a small residual range.

Although potential artifacts may result in waxing and waning of species ranges and diversity at the clade level^46^, our empirical data sets differ from the predictions of random models of clade evolution, in conforming to the predictions of higher levels of sympatry, hence high incidence of specialization, occurs during the late portion of a clade existence.

Weak directionality theory provides a consistent and widely applicable explanation for the extinction of clades. They diversify and survive because individual species become more and more specialised over time. Yet, the very reason for clade success also contains the seed of their decline. The path to extinction is neither simple nor monotonic. We identified more shift points than clades, implying that clades may survive moments of crisis. It will be interesting to know how phenotypic diversity evolves during clade existence, in order to understand whether phylogenetic or developmental constraints on evolvability might prevent clades from escaping the evolutionary trap that weak directionality represents^47^,^48^.

## Methods

We retrieved from the Paleobiology Database (https://paleobiodb.org/#/) data on fossil occurrences for 58 clades of marine invertebrate species selecting the fields occurrences, “advanced options”, taxonomic resolution: “species”, Plate: “Scotese”, output options: “collection”, “coordinates”, “methods”, “paleolocation” (see supplementary information for full details). Each data point includes the paleocoordinates and the (estimated) minimum and maximum age of the fossil localities. Data pertain to five different animal phyla (Arthropoda, Brachiopoda, Bryozoa, Cnidaria, Mollusca) and cover some 480 million years of the fossil record. Overall, the database includes ca. 21,000 species. The fossil record of individual clades was divided in equal-length time bins. The length of such intervals was clade-specific, meaning that we applied bins of different lengths as to maintain as many bins as possible while avoiding producing bins containing less than three species with at least three occurrences per species, which is the minimum requirement to calculate a species range size estimate. We further removed the species and genera that lack a continuous stratigraphic range and dubbed with uncertain taxonomic classification (e.g. sp., cf.). After applying these selection criteria, we were left with 21 clades, including 14,431 species and 84,457 occurrences overall.

### Species and clades range size estimation

For each species in each time bin, we estimated the range size (in km^2^) by computing its Minimun Convex Polygon (MCP^49^), under the software Quantum GIS 2.10^50^. Because of MCP represents the extent of occurrence of a species, land portions could be included in a marine species’ geographic range erroneously. In this very case, we removed land portions from the MCP by using the digitized version of 19 world maps displaying the reconstructed position of landmasses and seas (http://cpgeosystems.com/index.html) in the temporal interval from 550 to 70 Myr. In particular, when we had species geographic extent ranging less than 180 decimal degrees in longitude, we used the Lambert Azimuthal Equal Area projection. In all other cases, when we had species extent exceeding 180 decimal degrees of longitude and included within + −60 latitudinal degrees, we used Mollweide Equal Area projection. If the longitudinal extent exceeded 180 decimal degrees and latitudinal extent exceeded 60 decimal degrees north or south to the equator, we then used the Albers Equal Area projection. In the very special cases not considered in the above criteria we splitted the polygons in order to have different regions to be projected by means of Lambert Azimuthal Equal Area projection and then summed the areas computed for every single polygon portion to get the original species polygon area.

**Figure 1:**
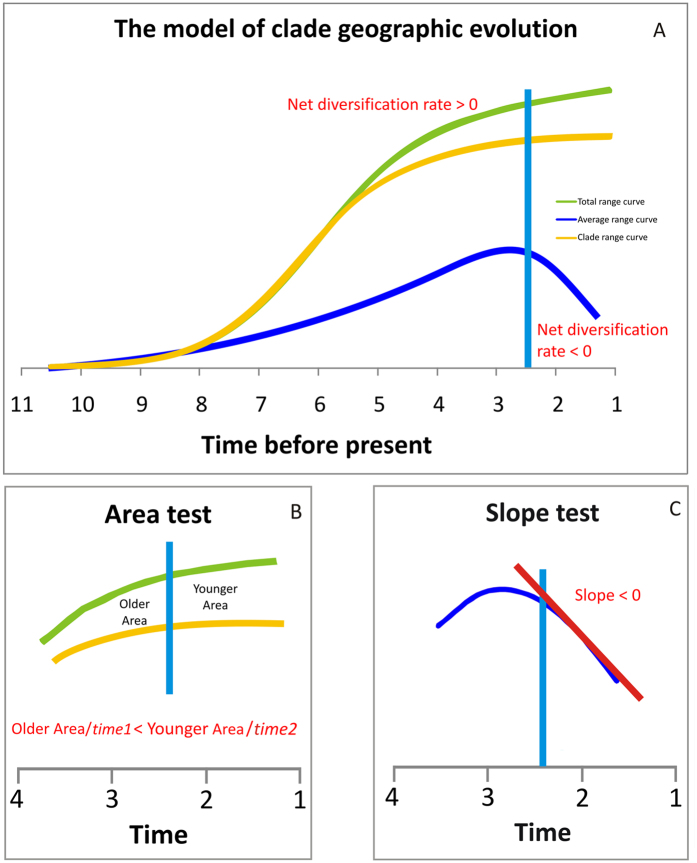
The weak directionality theory of clade geographic evolution. (**A**) The total (green solid line), average (blue solid line), and clade (gold solid line) range size curves for the focal clade. The total range curve is computed as the algebraic sum of individual species range sizes over time. The average range curve is computed dividing the total curve for the number of species present in each time bin. The clade range curve represents the range actually occupied by the entire clade, summed over consecutive time bins. According to weak directionality theory predictions, after the shift point (vertical light blue line) the total- and the clade range curves should diverge signficantly over time, as an effect of a progressive increased range overlap (sympatry). The area test (**B**) is devised to test such prediction. As species range sizes are expected to decrease, on average, after the shift points, the average range curve should take a negative slope after the shift (**C**). The slope test is devised to test such prediction.

**Figure 2:**
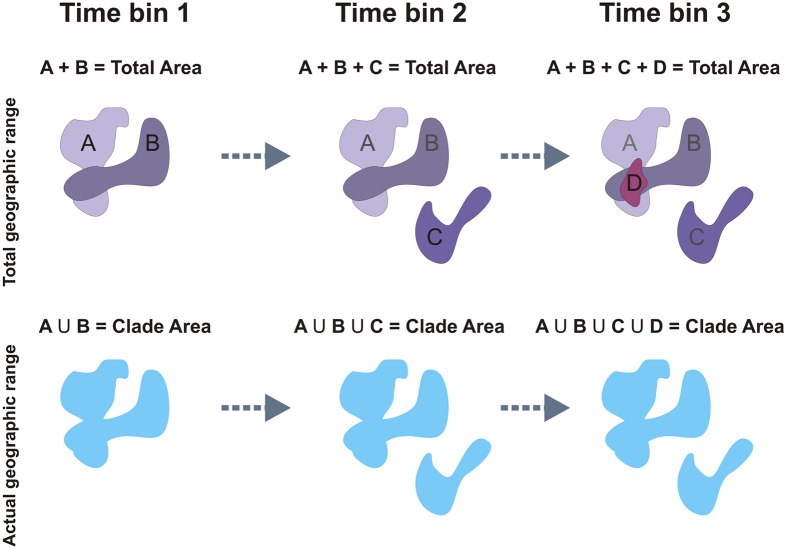
Difference between total and clade range curves computation. The total geographic range is computed by summing the range of each species in each time interval and then over successive intervals. The actual range is the real range of the clade, thus it is computed as the union of species’ areas, subsequently summed over consecutive time bins. In the figure, the shaded areas represent the species ranges. Species are indicated by capital letters. For each time bin, extinct species are indicated in grey color, living species are reported in black. Upper row: computation of the total range curve. Lower row: computation of the clade range curve.

After computing individual species ranges, we summed them to get the total range in the bin. Time-bin total species ranges were then summed over consecutive bins to get the total range curve (Fig. 1A, green line). For each bin and clade, we also computed the clade range (the range effectively occupied by the entire clade in the focal bin, Fig. 2). Clade ranges were summed over consecutive bins as well, to get the clade range curve (Fig. 1A,B gold line). The difference between the actual and the total ranges per bin depends on how much species ranges overlap to each other (i.e. on the degree of sympatry).

Finally, we divided the total range per bin by the number of species present in that bin. This gives the average species range size (Fig. 1A,C blue line) of the species of that particular clade during that particular time bin.

### Diversification rate analyses using PyRate

We analyzed fossil occurrence data for each clade using the program PyRate^51^,^52^, which provides a joint Bayesian estimation of the preservation process and diversification dynamics. We modeled fossil preservation by a non-homogeneous Poisson process with rate estimated from the data and expressing the mean number of expected occurrences per lineage per time unit (in this case 1 Myr). Under the PyRate framework, the preservation process is used to infer times of origination and extinction (when applicable) of all lineages, which represent the result of an unknown underlying birth-death process. The parameters of the birth-death process, speciation and extinction rates, represent the expected number of speciation/extinction events per lineage per Myr, and are estimated from the data along with the times of origination and extinction and preservation rate. We ran 10,000,000 Markov Chain Monte Carlo (MCMC) iterations to obtain posterior estimates of the parameters. We assessed the presence of significant shifts in speciation and/or extinction rates through time, using the birth-death MCMC algorithm^52^. Based on the estimated number of shifts and their temporal placement, we obtained marginal posterior distribution of speciation, extinction, and net diversification rates calculated within 1 Myr time bins through the lifespan of each clade. Finally, after discarding the initial 2,000,000 iterations as burnin, we summarized rates through time by calculating mean and 95% credible intervals from the posterior samples, and used them to produce rates-through-time plots.

#### Statistical testing of weak directionality theory

Shiftpoints estimation and testing: We identified shift points (in time) by using the cross-entropy method, which applies the Bayesian information criterion to locate significant changes in the trend along the net diversification, total range, and clade range curves^53^. Then, we computed the mean age distance among the three. We compared such mean distance to a family of random mean age distances, to test whether the sum of the time distances among them was smaller than expected by chance, which would imply the shift points are statistically coincident in time. To perform such comparison, we sampled at random 9,999 times two ages from time-bins midpoints (the degree of sympatry and cumulative total range curves are computed per time-bin), and one age from the net diversification rate, 1-my long sample. Then, we calculated the mean distance among the three random time points. Eventually, we counted how many times the random mean time distance is higher than the real distance between breakpoints, to calculate the p-value for the hypothesis that real time distances are smaller than random means.

Testing the increase in degree of sympatry after the shift point: After locating the shift points, we first tested whether the degree of sympatry increases after them (the area test, see Fig. 1B). To this aim, we computed the area difference (i.e. the area between the two curves) between the cumulative total and the cumulative actual ranges, both for the total time intervening from clade birth to the shift point, and from the shift point to the moment of clade death. Then, we divided each area differences to the corresponding lengths of time. This gives two area differences per unit time. The ratio of the two differences, expressed as a percentage of the unit area difference after the shift point to the unit area difference before the shift point, indicates whether the degree of sympatry either increases or decreases towards the present, so that a value <100 points to a decrease, and a value >100 to an increase, in the degree of sympatry over time.

Testing for average range size reduction after the shift point: According to weak directionality theory, the species average range size should decrease after the shift points. To test such a prediction, we regressed species average range size against time, selecting only those time bins more recent than the shift points. A significant and negative regression slope would indicate the expected trend towards range size reduction (slope test, see Fig. 1C).

Testing for net diversification rate decrease after the shift point: We partitioned the per-million year net diversification rate, in rates before and rates after the shift points, and applied to the two samples Wilcoxon’s (Mann-Whitney) one-sided test, to asses the hypothesis that net rates significantly decrease after the shift points.

Testing for the correlation between diversification and the degree of sympatry: We used a time-varying birth-death model to investigate the intensity and significance of correlations between speciation and extinction rates and the degree of sympatry^52^. The model is implemented in PyRateContinuous (https://github.com/dsilvestro/PyRate) and assumes an exponential correlation between sympatry and the birth-death rates, parameterized by correlation parameters γ_λ_ and γ_μ_, which are estimated in a Bayesian framework. A posterior estimate γ > 0 indicates positive correlation between sympatry and the speciation (or extinction) rates, and γ < 0 indicates negative correlation. We considered the correlations as significant when 0 did not fall within their 95% posterior credible intervals. We used the times of origination and extinction of each species estimated by PyRate (see above) as input data for the PyRateContinuous analyses, and ran 1,000,000 MCMC iterations sampling every 1000^th^, to obtain posterior samples of the correlation parameters and baseline speciation and extinction rates. We calculated the posterior means and 95% credible intervals of the correlation parameters after removing the first 200 samples as burnin.

## Additional Information

**How to cite this article**: Raia, P. *et al*. Progress to extinction: increased specialisation causes the demise of animal clades. *Sci. Rep.* **6**, 30965; doi: 10.1038/srep30965 (2016).

## Supplementary Material

Supplementary Information

## Figures and Tables

**Table 1:** Likelihoods obtained by comparing total range size cumulative curves to three different theoretical curves corresponding to the linear, sigmoid, and generalized logistic models.

Clade	linear	sigmoid	generalized logistic	best model
Desmoceratidae	2.337	7.935	8.082	generalized logistic
Auloporida	2.422	7.695	7.240	sigmoid
Cystiphyllida	2.296	7.724	7.121	sigmoid
Favositida	2.577	9.402	8.504	sigmoid
Stauriida	2.799	9.899	9.477	generalized logistic
Pterineidae	2.321	6.510	7.065	sigmoid
Athyridida	2.697	8.800	9.365	generalized logistic
Orthida	2.675	8.743	9.327	generalized logistic
Orthotetida	2.579	9.356	8.954	sigmoid
Productida	2.586	8.839	9.360	generalized logistic
Spiriferida	2.581	8.996	9.512	generalized logistic
Spiriferinida	2.655	9.684	9.087	sigmoid
Strophomenida	2.397	9.263	8.444	sigmoid
Cystoporida	2.393	8.439	7.355	sigmoid
Fenestrida	2.347	8.396	7.607	sigmoid
Rhabdomesida	2.325	5.947	6.731	sigmoid
Trepostomida	2.446	8.260	8.032	sigmoid
Bellerophontidae	2.272	8.186	7.975	sigmoid
Euomphalidae	2.252	6.749	7.569	sigmoid
Lophospiridae	2.328	8.773	7.845	generalized logistic
Proetidae	2.289	7.344	8.113	sigmoid

Comparisons were made by means of maximum likelihood estimation. Log-likelihoods are reported. The last column indicates the best fitting model based on AICc.

**Table 2:** Average age of the shiftpoints (in Ma) and the percentage of time since clade inception to the shiftpoints.

Major Clade	clade	shiftpoints	p.dist	area.test	net.t	slope	p.slope
Ammonites	Desmoceratidae	101.7 (56.8%)	**0.001**	**0.68**	**0**	−10941.1	0.659
Anthozoans	Auloporida	393.9 (28.5%)	**0.007**	136.64	**0**	−1486.7	0.059
Anthozoans	Auloporida	288.3 (85.6%)	**0.024**	504.43	**0**	9156.0	0.151
Anthozoans	Cystiphyllida	393.1 (84.5%)	0.184	**0**	**0**	164389.6	NA
Anthozoans	Favositida	415.5 (17.8%)	**0.003**	**30.98**	**0**	−9464.8	**0.000**
Anthozoans	Favositida	383.1 (34.2%)	**0.002**	**32.11**	**0**	−1562.2	**0.012**
Anthozoans	Stauriida	358.8 (47.7%)	1.000	**16.88**	**0**	−2772.6	**0.000**
Anthozoans	Stauriida	361.2 (46.5%)	0.199	**18.05**	**0**	−2772.6	**0.000**
Anthozoans	Stauriida	372.9 (40.7%)	1.000	**23.38**	**0**	−2810.6	**0.000**
Bivalves	Pterineidae	400.5 (27.2%)	**0.046**	**25.09**	**0**	−4884.8	**0.046**
Brachiopods	Athyridida	306.4 (55.3%)	0.870	**13.55**	**0**	−2118.5	**0.014**
Brachiopods	Athyridida	284.9 (63.3%)	0.298	**12.78**	**0**	−810.1	0.325
Brachiopods	Orthida	372.8 (50.2%)	0.780	**53.97**	**0**	−1040.8	**0.000**
Brachiopods	Orthida	360.2 (55.4%)	0.512	**49.33**	**0**	−916.3	**0.011**
Brachiopods	Orthotetida	404.7 (24.9%)	**0.003**	**14.79**	1	−951.8	0.251
Brachiopods	Orthotetida	347.4 (53.2%)	**0.002**	**22.37**	0.974	886.4	0.601
Brachiopods	Orthotetida	294.7 (79.2%)	**0.000**	**13.73**	**0**	8810.1	0.211
Brachiopods	Productida	276.9 (87.1%)	**0.038**	**0.03**	**0**	18956.9	0.144
Brachiopods	Spiriferida	316.9 (64.6%)	1.000	**45.61**	**0.014**	453.7	0.732
Brachiopods	Spiriferinida	258.3 (38.0%)	**0.016**	**0.909**	**0**	219.1	0.335
Brachiopods	Strophomenida	406.7 (43.0%)	**0.004**	**61.31**	**0**	−6006.0	0.068
Bryozoans	Cystoporida	276.5 (86.5%)	**0.021**	**0.69**	**0**	48723.2	0.093
Bryozoans	Fenestrida	292 (78.3%)	**0.000**	**0.08**	**0**	−4184.0	0.612
Bryozoans	Rhabdomesida	294 (79.8%)	**0.021**	**1.67**	**0**	−10506.9	**0.008**
Bryozoans	Trepostomida	399.4 (19.9%)	**0.000**	**34.08**	**0**	1655.2	0.003
Bryozoans	Trepostomida	274.2 (59.8%)	**0.003**	**44.81**	**0**	2291.5	0.023
Gastropods	Bellerophontidae	324.2 (66.0%)	**0.000**	**1.09**	**0**	4228.9	0.046
Gastropods	Euomphalidae	356.7 (48.8%)	**0.000**	**74.53**	**0**	−5382.5	**0.003**
Gastropods	Lophospiridae	294.3 (62.4%)	**0.059**	**82.41**	**0**	−4851.4	**0.004**
Trilobites	Proetidae	375.2 (58.0%)	**0.045**	**58.74**	**0**	−22289.3	0.148

The probability that the distance among shift points is lower than expected by chance (*p.dist*). The ratio of the percentage of area per unit time between the total range and the clade range curves after the average shiftpoint as compared to the same figure before the shiftpoint (see Fig. 1 for further explanation)(*area.test*). The probability that net diversification rates decrease after the shiftpoint (*net.t*). The slope of the regression between the average species range and time after the shiftpoint (*slope*) and the probability that such a regression slope is different from zero (*p.slope*). Values in bold indicate compliance to weak directionality theory.

**Table 3:** The distribution of positive cases (i.e. either in accordance or not with the hypotheses tested) for the clade statistics reported in Table 2.

	p.dist	area.test	net.t	slope.test
% significant shifts	66.67	93.33	93.33	80
p.value (binomial test)	0.0280	≪0.0001	≪0.0001	0.0351
% significant clades	76.19	95.24	1	66.67
p.value (binomial test)	0.0279	≪0.0001	≪0.0001	0.1890

The statistical comparison is made according to the binomial distribution, given a priori success ratio of 0.5. Binomial tests are computed for both individual shiftpoints, and for clades. For the latter each hypothesis is assumed to be verified if at least one shiftpoint complies with the predictions. Values in bold indicate compliance to weak directionality theory.

**Table 4:** Correlation between the degree of sympatry and speciation and extinction rates.

Clade	Correlation with speciation (γ_λ_)			Correlation with extinction (γ_μ_)		
	mean	95% CI		mean	95% CI	
Athyridida	−**0.96**	−**2.05**	−**0.08**	**3.32**	**2.48**	**4.24**
Auloporida	7.84	2.49	13.90	1.01	−4.98	6.98
Bellerophontidae	−**3.15**	−**4.56**	−**1.50**	−3.54	−5.13	−2.08
Cystiphyllida	−2.35	−5.70	1.32	**7.44**	**4.55**	**10.14**
Cystoporida	−**5.79**	−**8.10**	−**3.77**	**1.62**	**0.12**	**2.97**
Desmoceratidae	−**6.15**	−**10.24**	−**1.97**	2.47	−2.09	6.63
Euomphalidae	−**3.64**	−**6.06**	−**1.08**	−0.05	−2.80	3.07
Favositida	−**1.61**	−**2.39**	−**0.66**	**3.75**	**2.55**	**4.83**
Fenestrida	−**3.29**	−**4.25**	−**2.15**	**3.25**	**2.01**	**4.35**
Lophospiridae	5.06	2.83	7.99	−4.11	−7.46	−0.04
Orthida	−**0.61**	−**0.91**	−**0.27**	**1.60**	**1.23**	**1.96**
Orthotetida	−**2.67**	−**3.85**	−**1.52**	**2.74**	**1.95**	**3.44**
Productida	−**0.80**	−**0.96**	−**0.65**	**0.82**	**0.64**	**0.99**
Proetidae	−**29.67**	−**35.09**	−**22.79**	−14.17	−22.02	−7.70
Pterineidae	−17.30	−38.02	0.95	−16.96	−37.90	3.15
Rhabdomesida	1.54	0.82	2.22	0.40	−0.42	1.07
Spiriferida	−**1.59**	−**2.34**	−**1.01**	**3.46**	**2.51**	**4.30**
Spiriferinida	−0.35	−1.22	0.57	**1.96**	**1.10**	**2.86**
Stauriida	0.64	0.41	0.85	**0.68**	**0.45**	**0.91**
Strophomenida	−0.43	−1.85	0.89	0.88	−0.47	2.18
Trepostomida	−**0.89**	−**1.22**	−**0.62**	**1.93**	**1.47**	**2.34**

Posterior sampled of the correlations parameters are summarized as mean values and 95% credible intervals (CI). The correlation parameters γ_λ_ and γ_μ_ quantify the correlation between temporal changes in the birth-death rates and changes in the degree of sympatry. For instance, the speciation rate at time *t* is λ_*t*_ = *λ*_*0*_ exp(γ_λ_ *s*_*t*_), where *λ*_*0*_ is the estimated baseline speciation rate and *s*_*t*_ is the degree of sympatry at time *t* (ref. 11). Values in bold indicate significant negative correlation with speciation and significant positive correlation with extinction rates.
