# Supplementary material for: Progress to extinction: increased specialisation causes the demise of animal clades
Source: Sci Rep. 2016 Aug 10;6:30965. doi: 10.1038/srep30965 (PMC4978992; doi:10.1038/srep30965)
Supplement: Supplementary Information [file srep30965-s1.doc]

**Progress to extinction: increased specialisation causes the demise of animal clades**

**Raia P.*^1^, Carotenuto F. ^1^, Mondanaro A. ^1^, Castiglione S. ^1^, Passaro F. ^1^, Saggese F. ^1^, Melchionna M. ^1^, Serio C. ^1^, Alessio L. ^1^, Silvestro D. ^2^, Fortelius M. ^3^**

**^1^** Department of Earth Science, Environment and Resources, University of Naples Federico II, 80138 Napoli, Italy.

**^2^** Department of Ecology and Evolution, University of Lausanne, 1015 Lausanne, Switzerland and Department of Biological and Environmental Sciences, University of Gothenburg, 413 19 Gothenburg, Sweden.

**^3^** Department of Geosciences and Geography, P.O.Box 64 (GustafHällströminkatu 2a), FIN-00014 Universityof Helsinki, Finland.

**Supplementary figures**

**Figure S1. For each individual clade, we report below the paths of the 1) cumulative ranges (upper left corner, the green line is the cumulative total range, the purple line is the cumulative actual range); 2) average species ranges (upper right corner); 3) net diversification rate (lower central, the gray shaded area represents the 95% confidence interval around the net diversification rate estimate).**

**
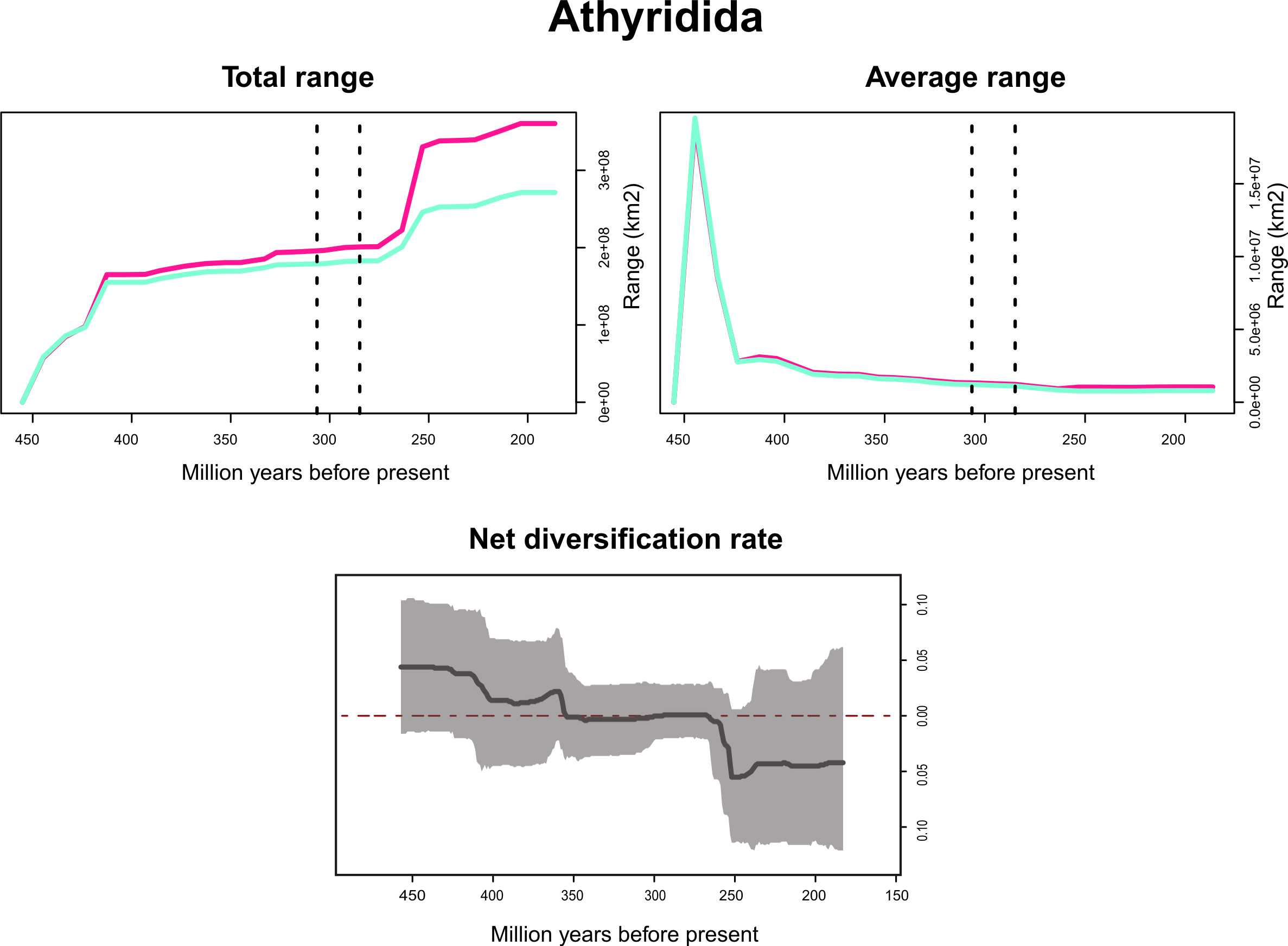

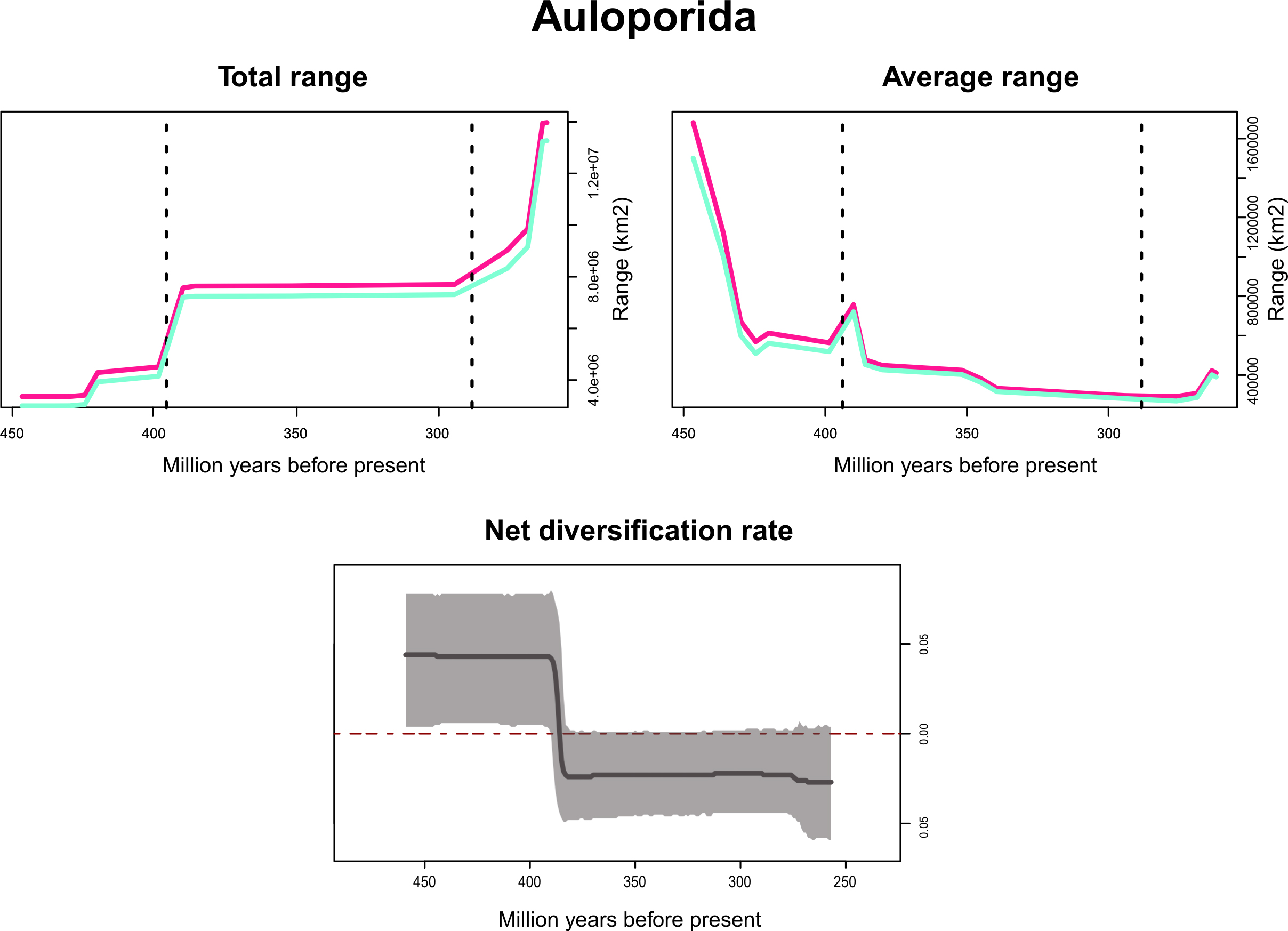

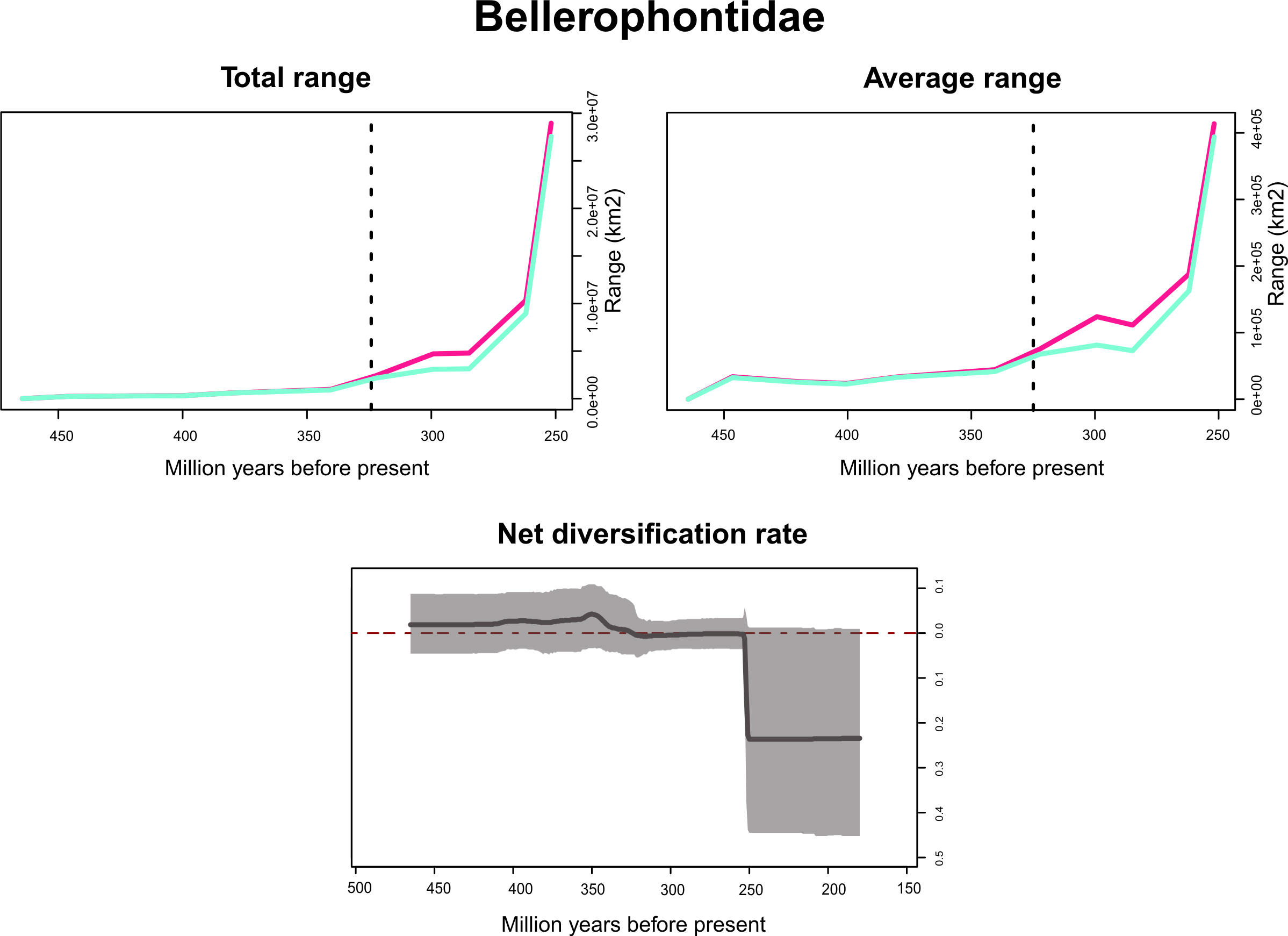

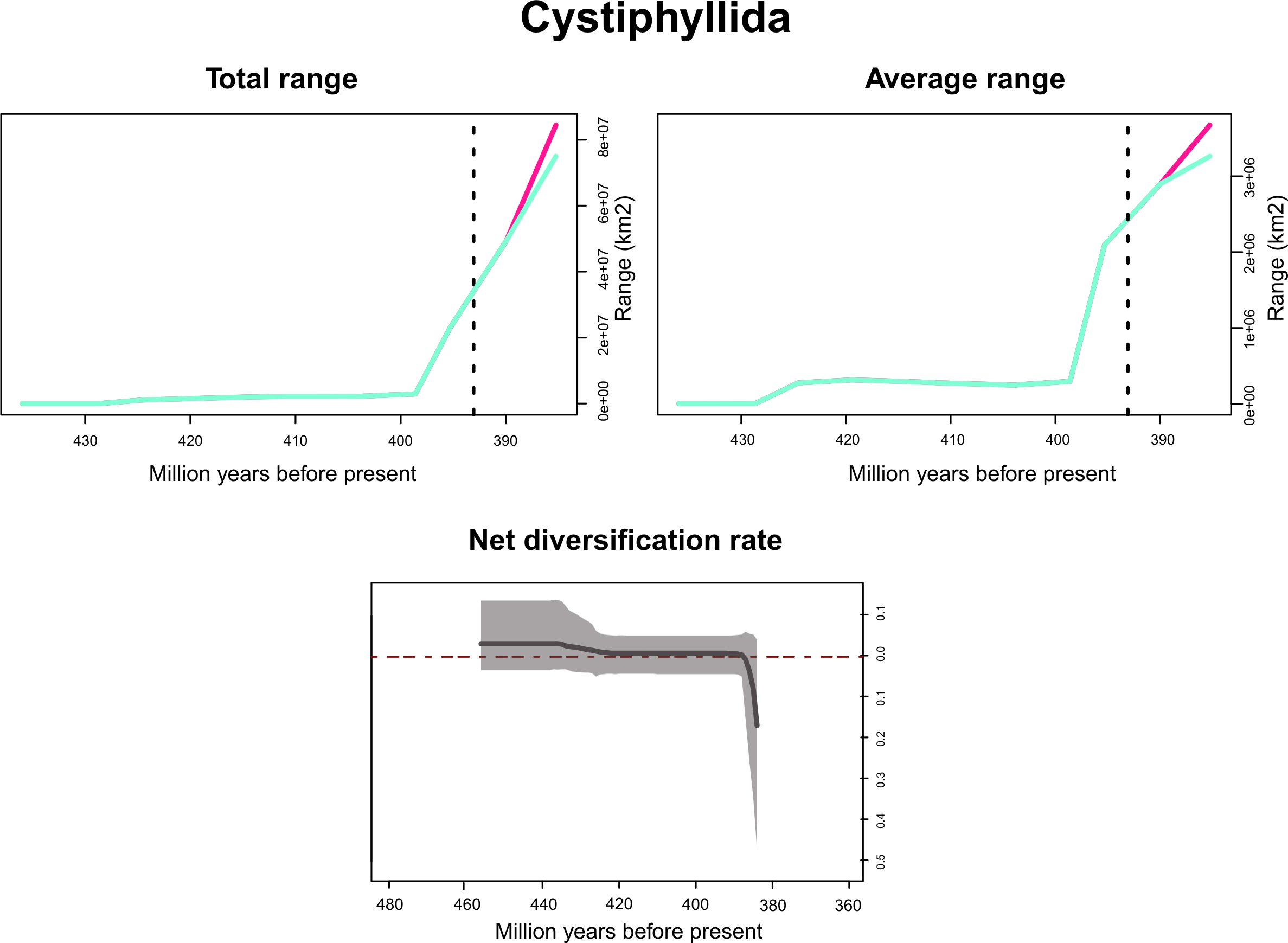

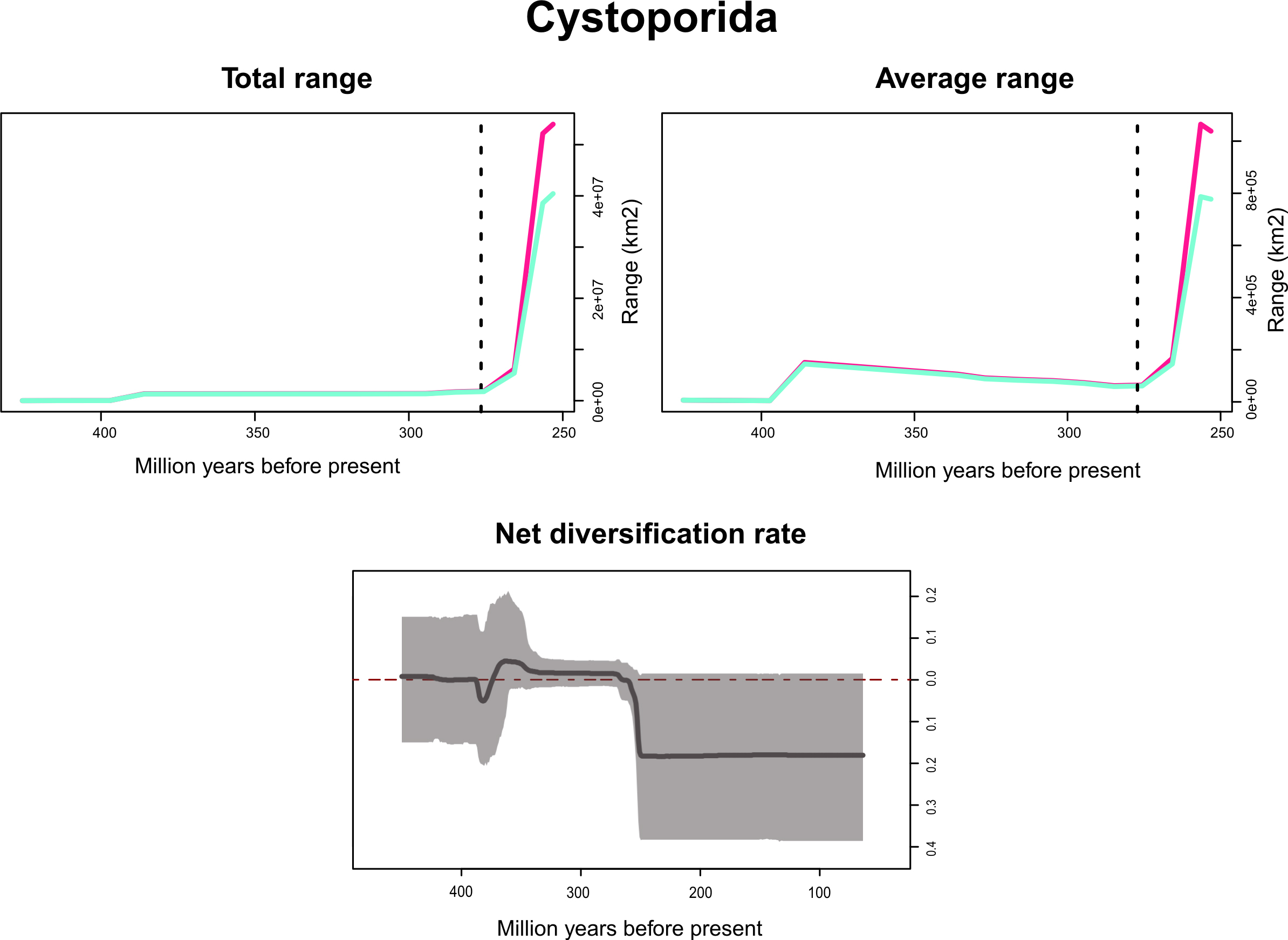

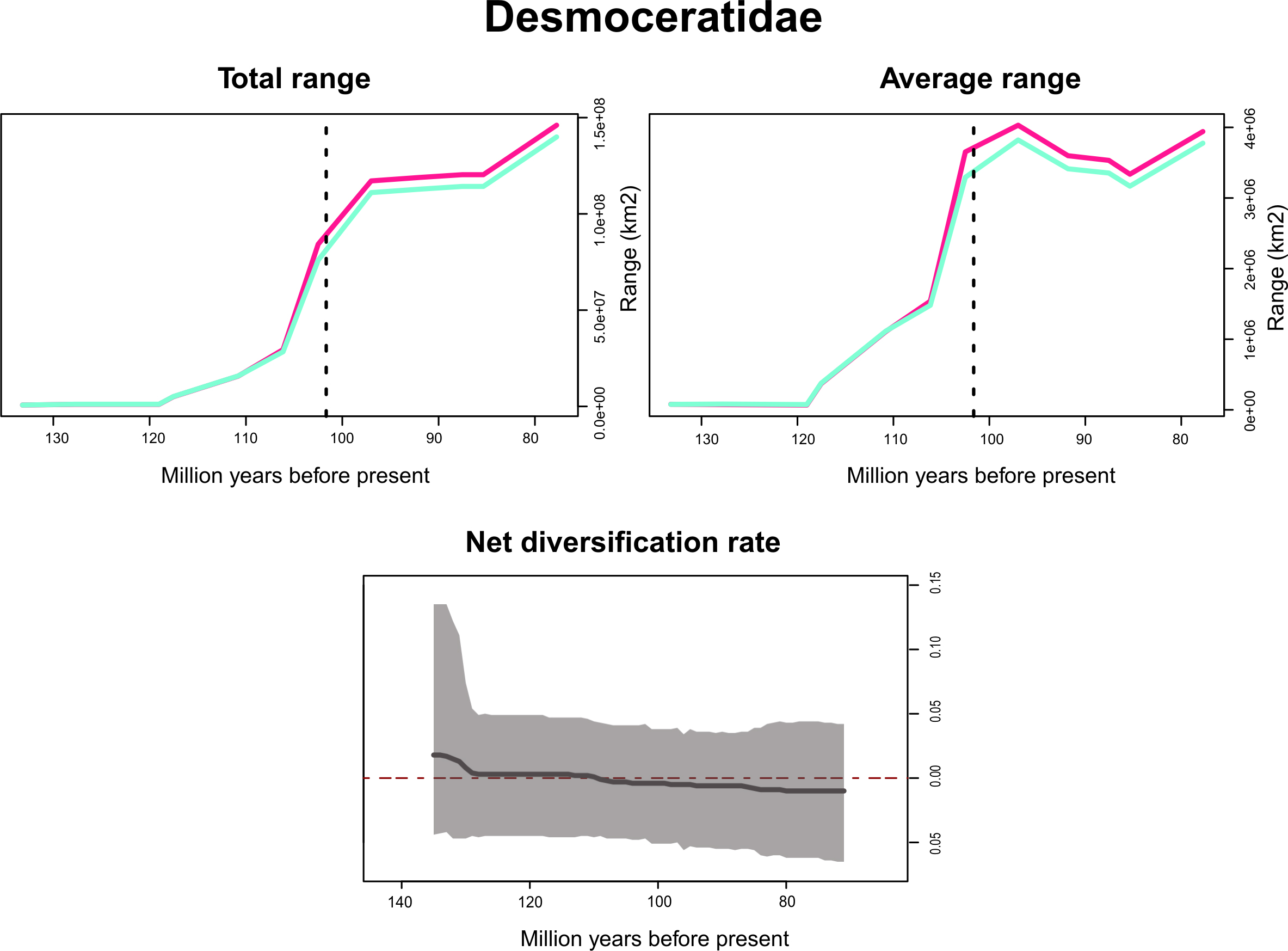

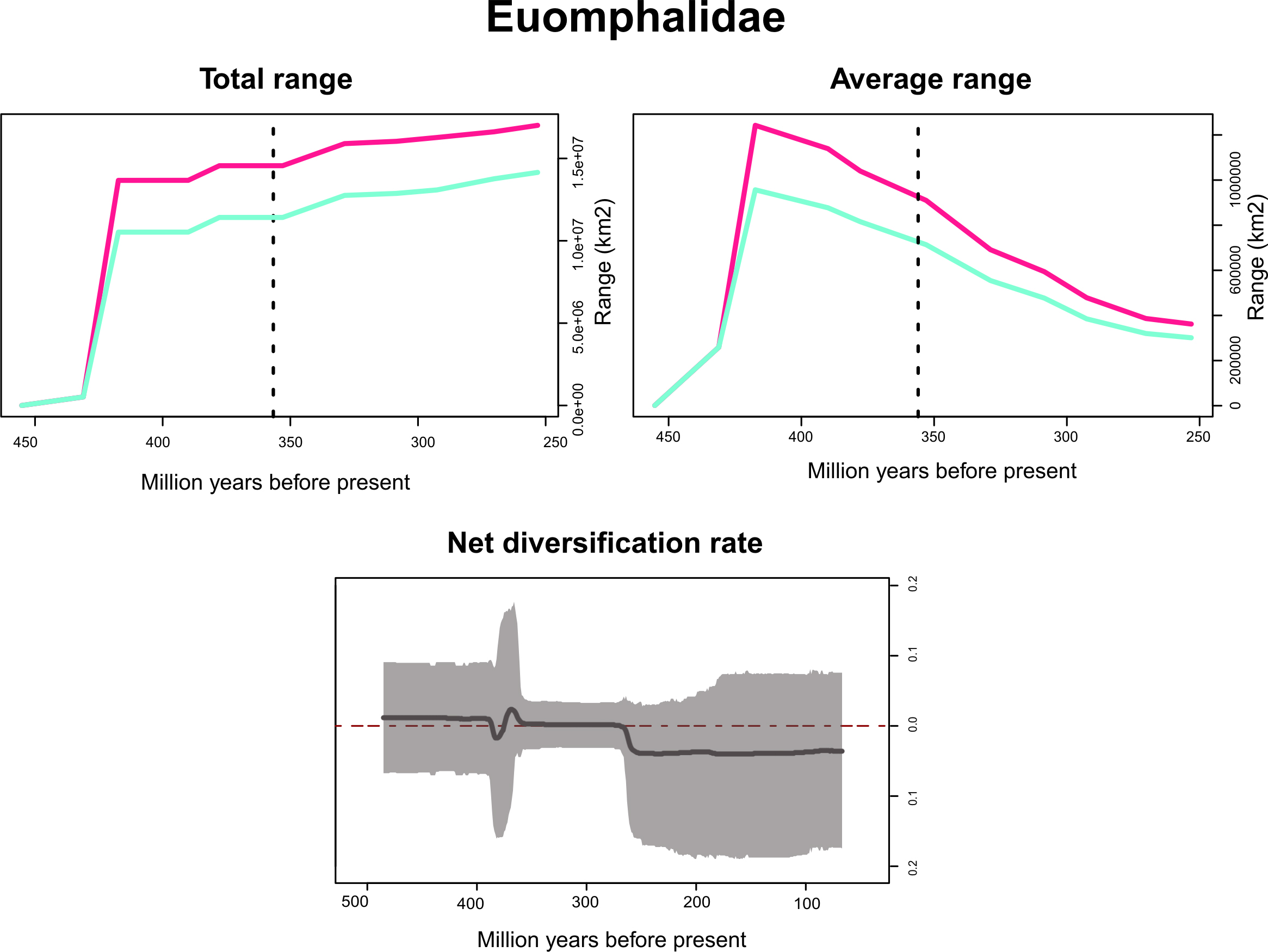

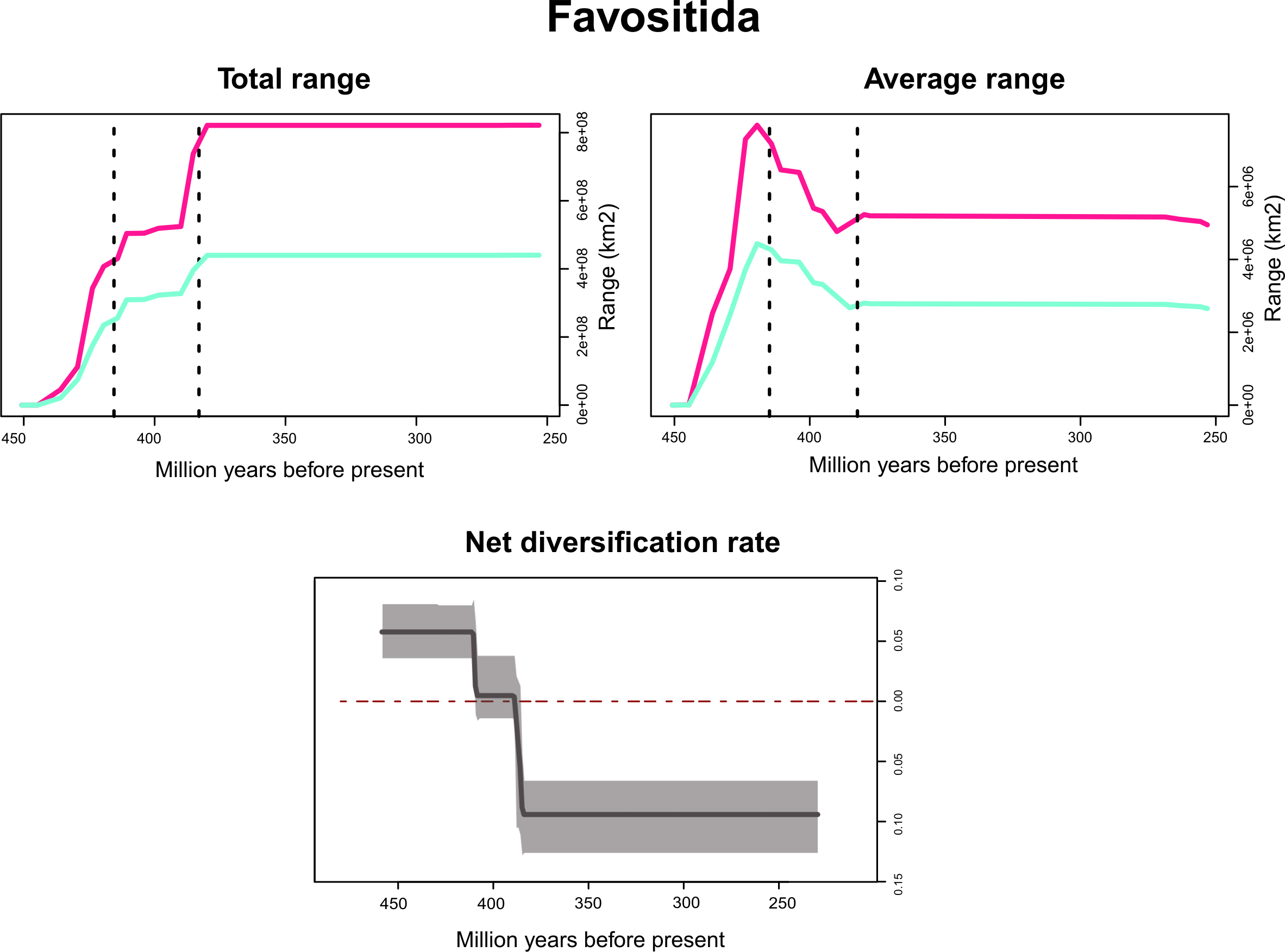

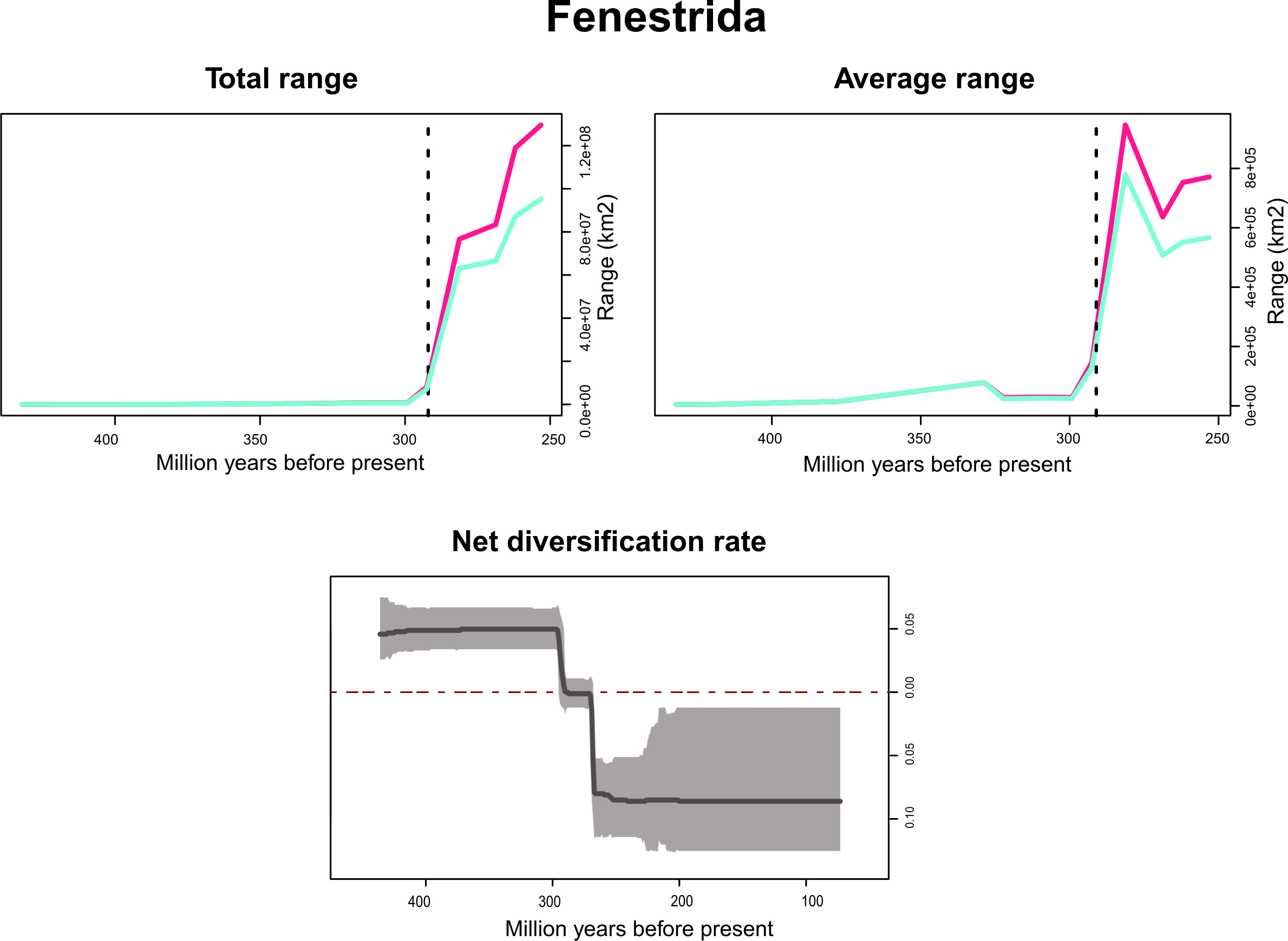

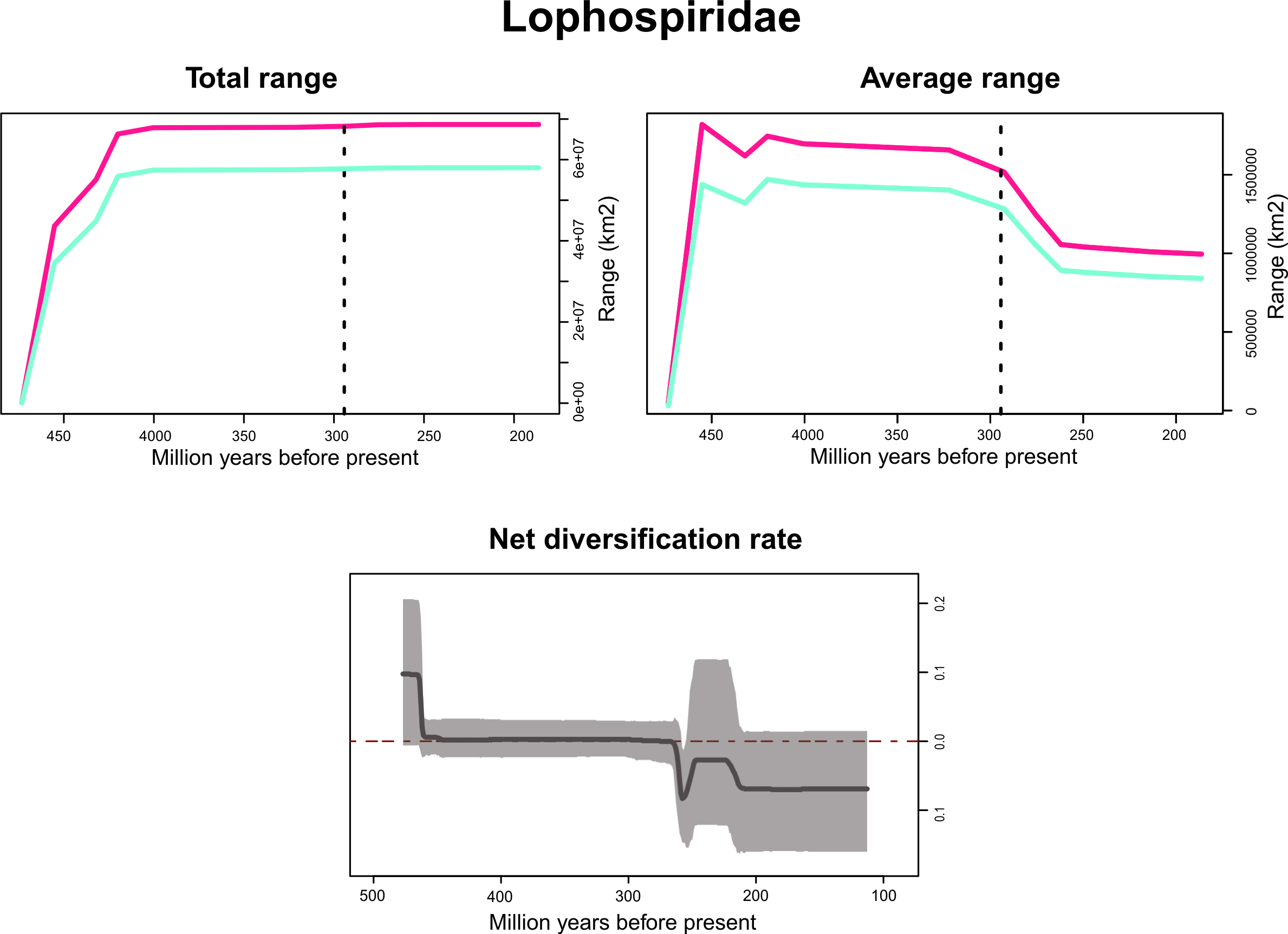

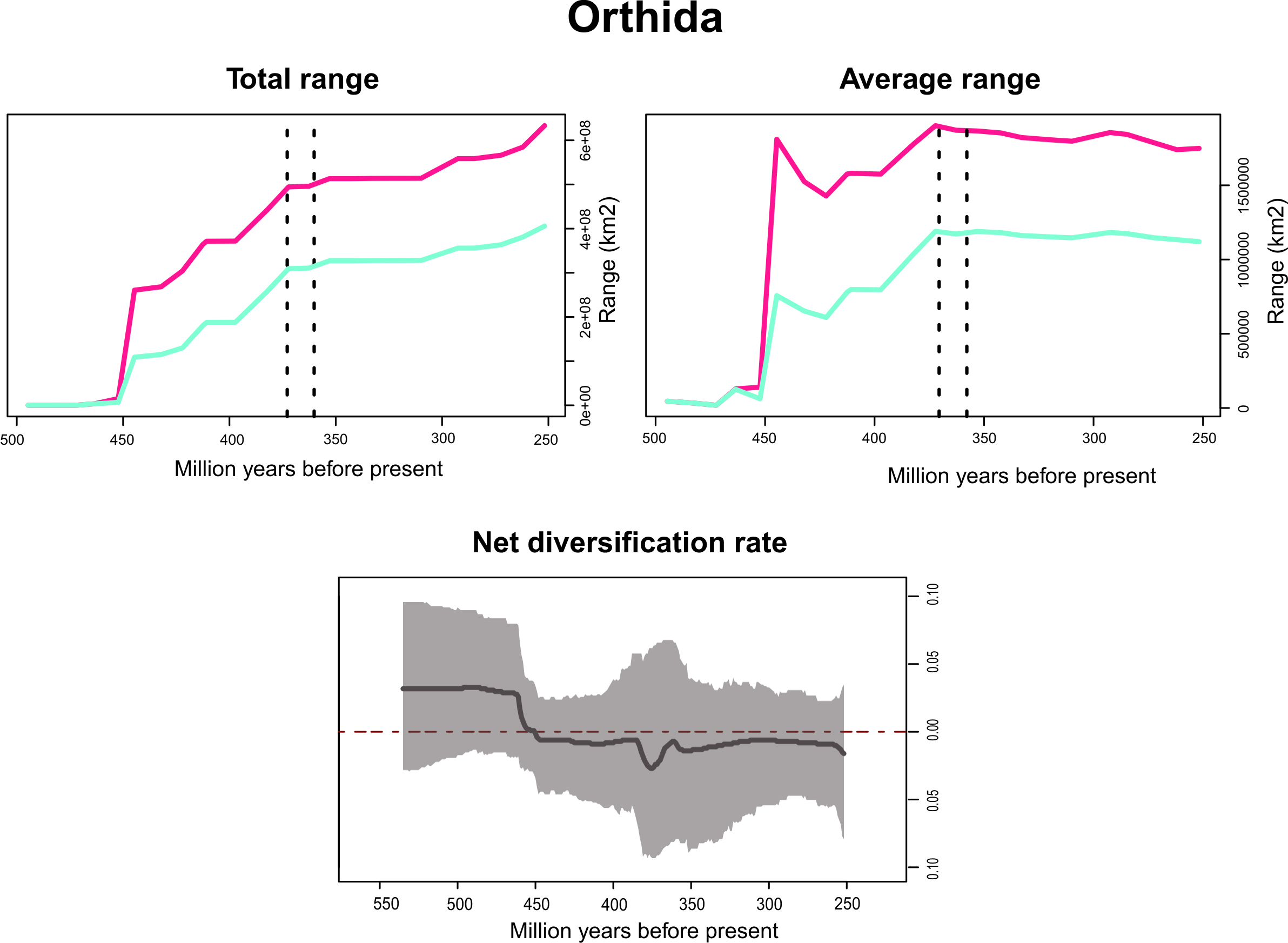

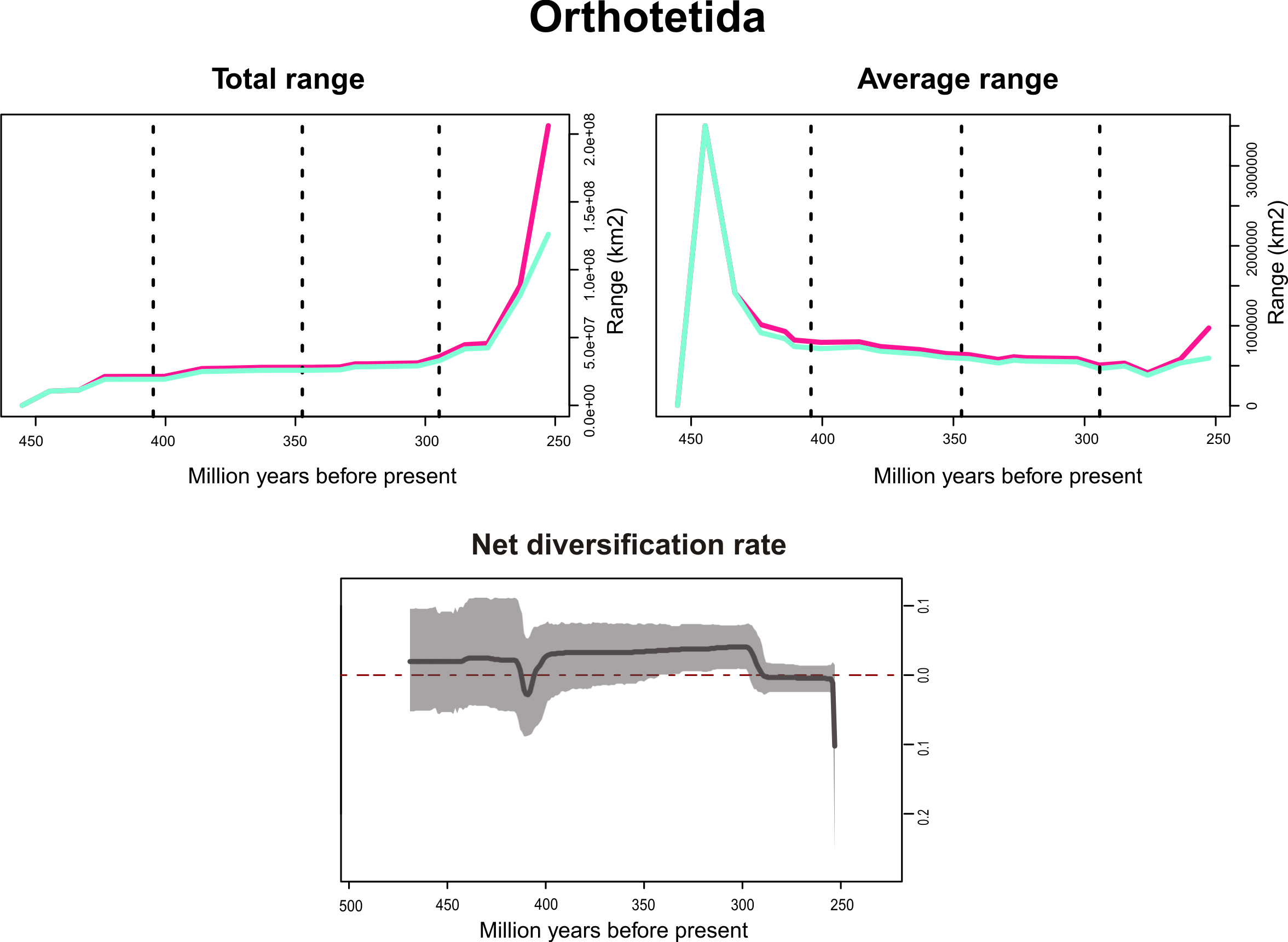

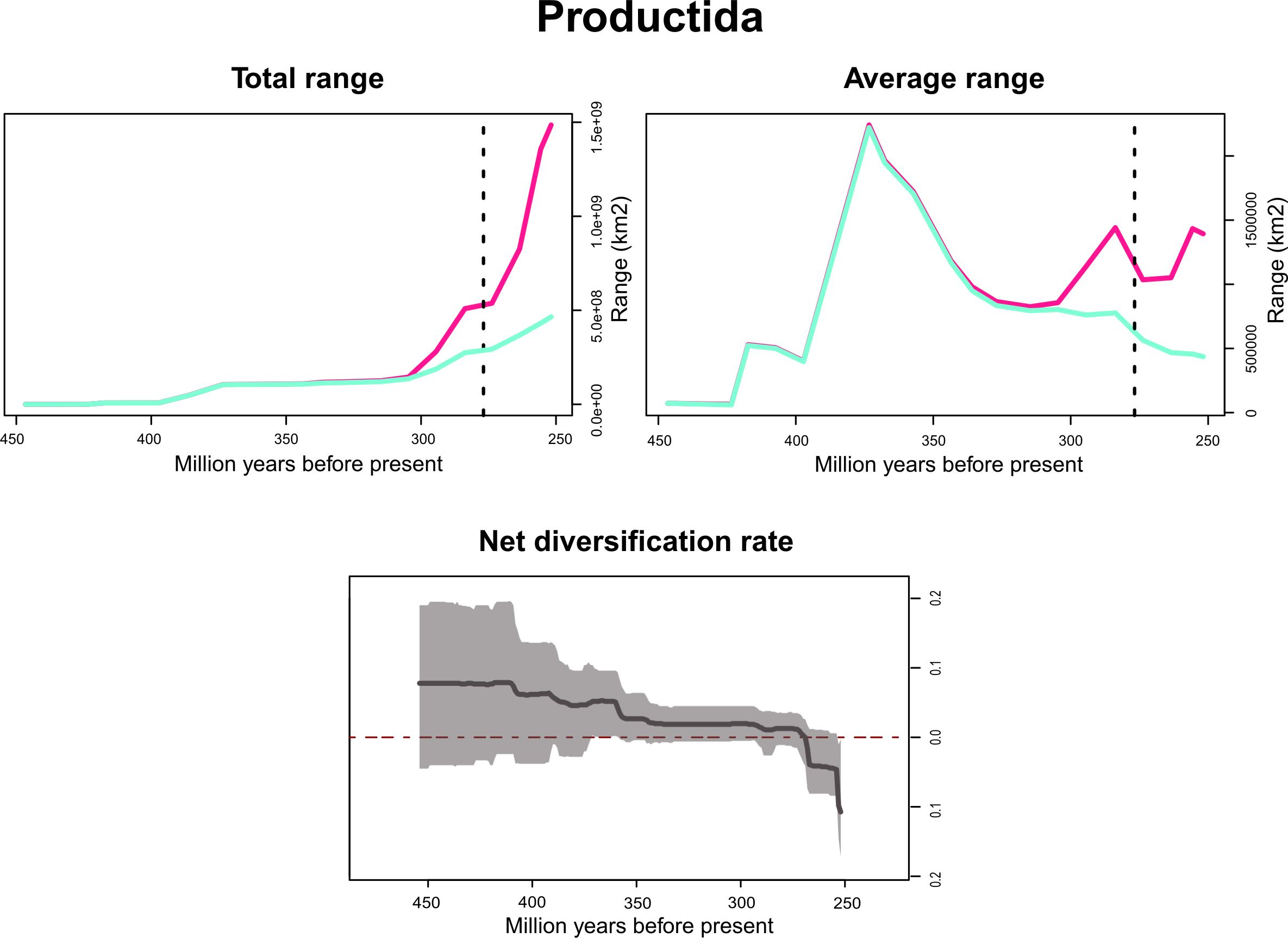

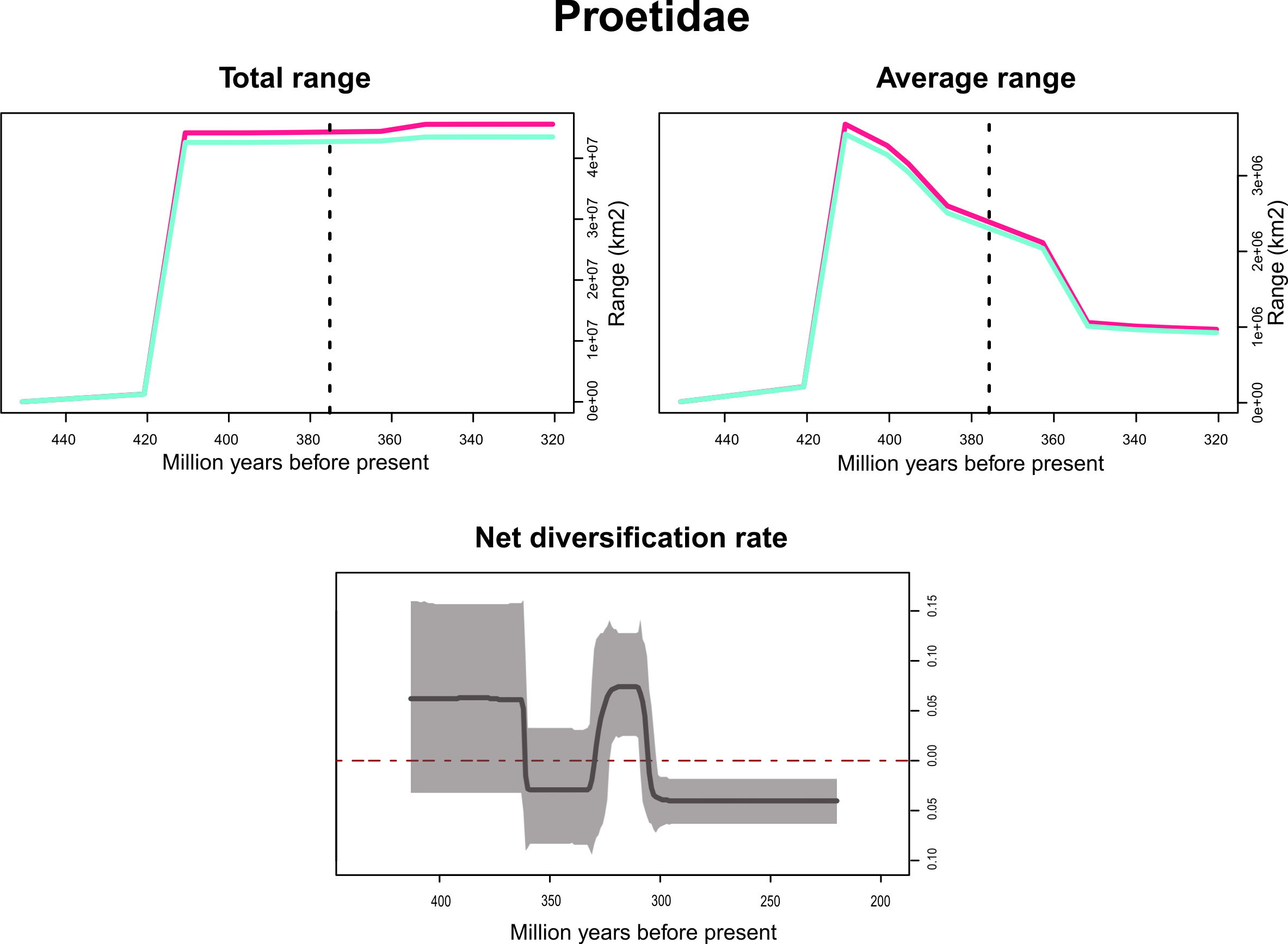

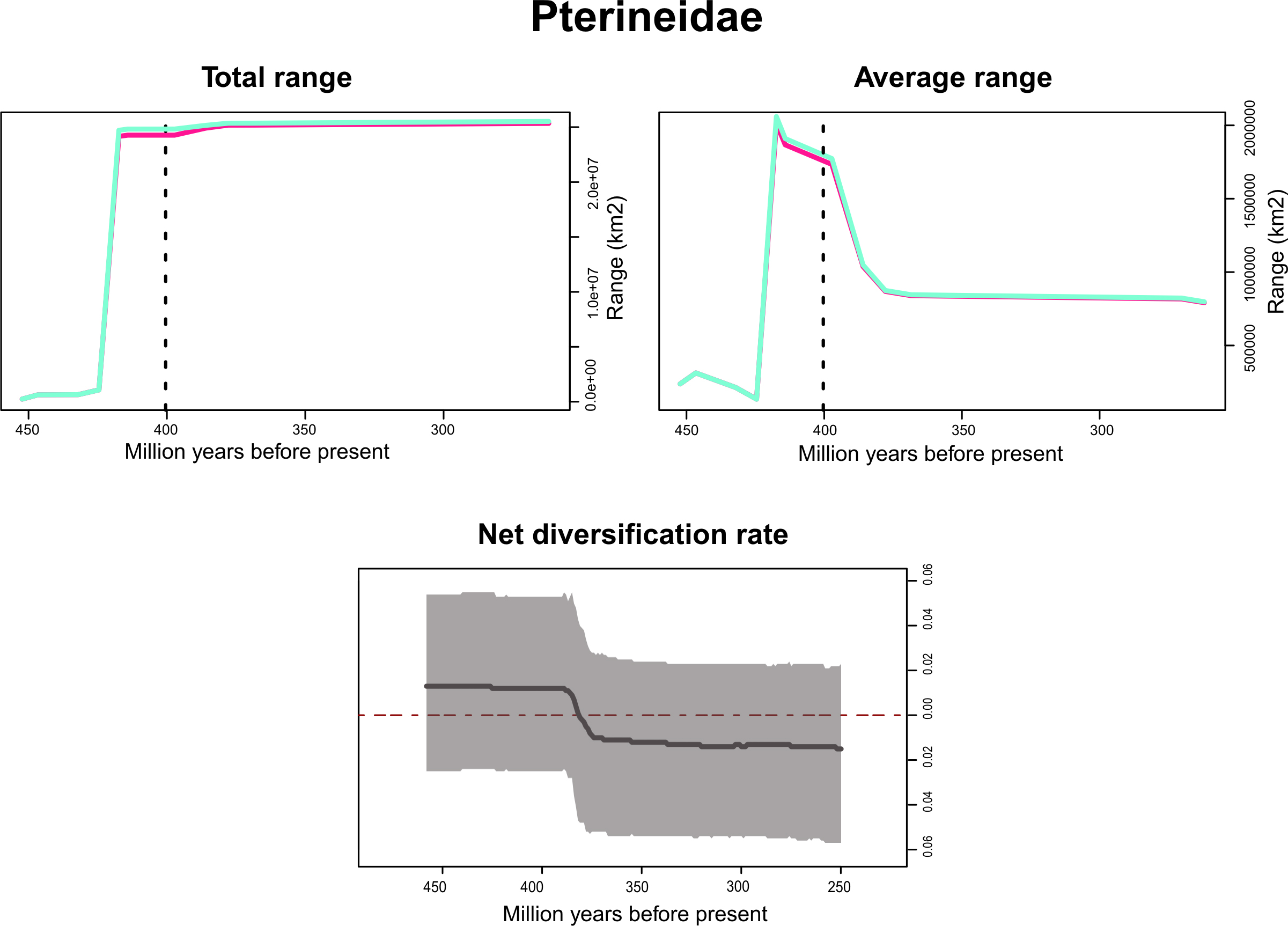

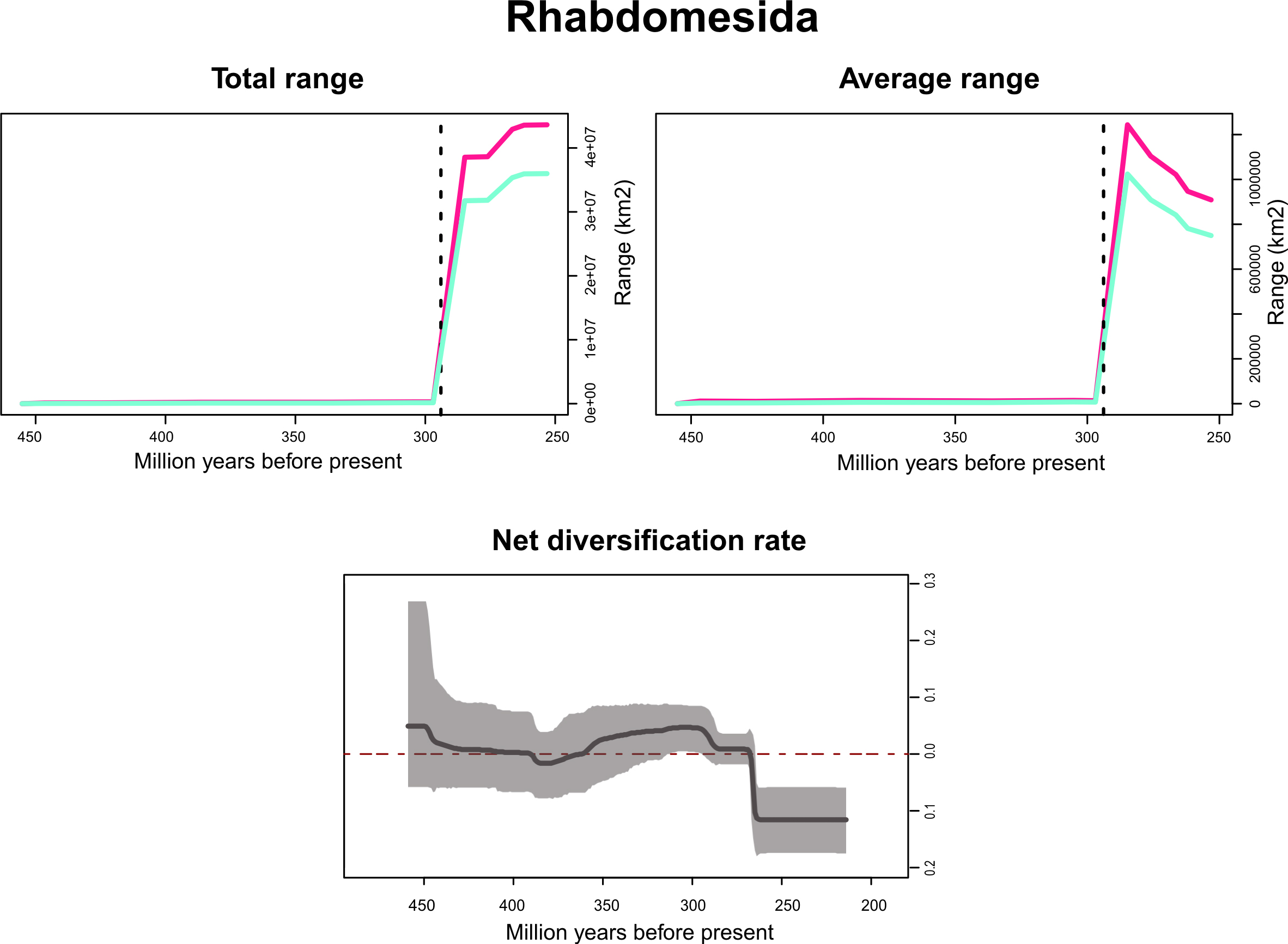

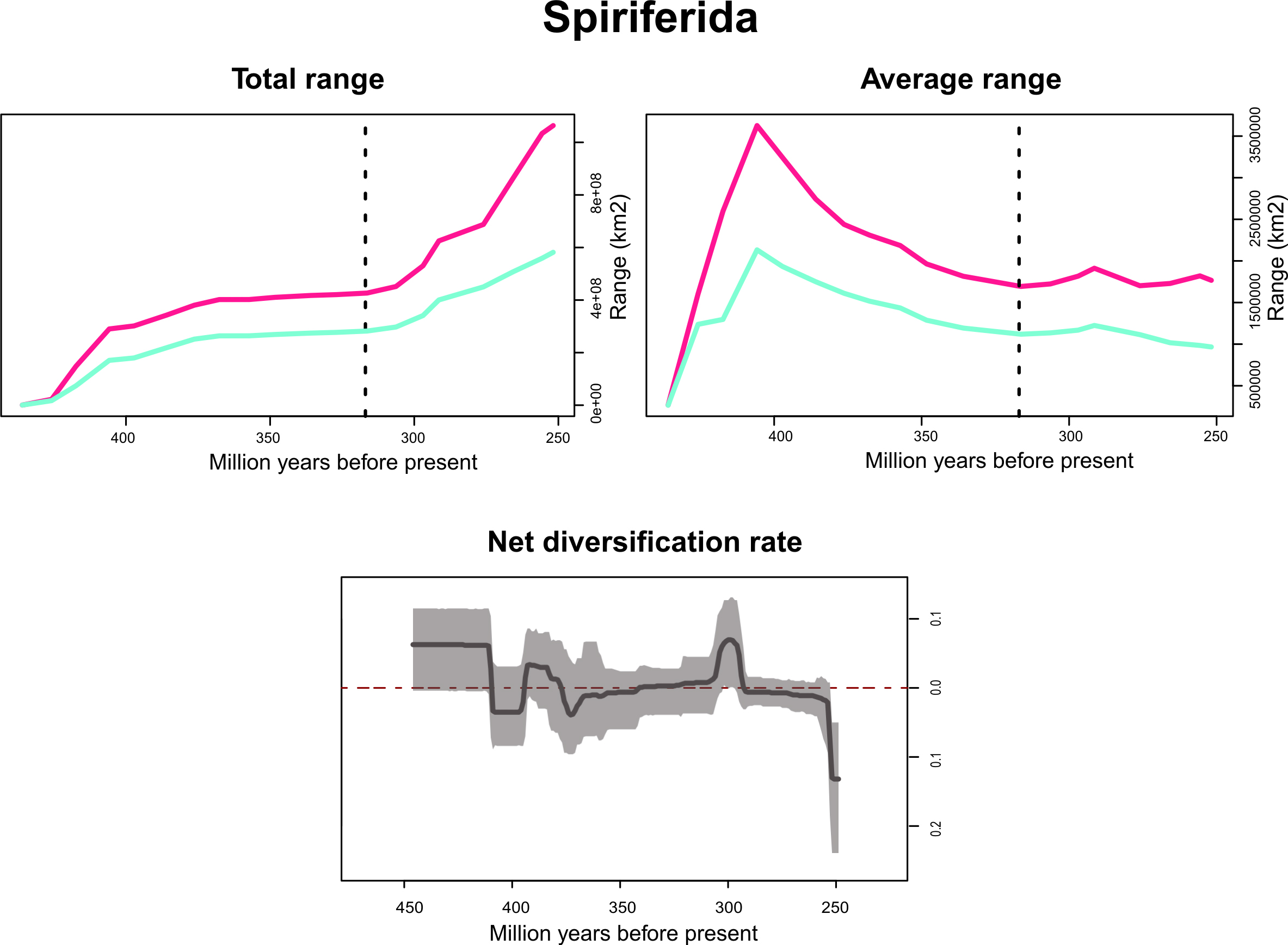

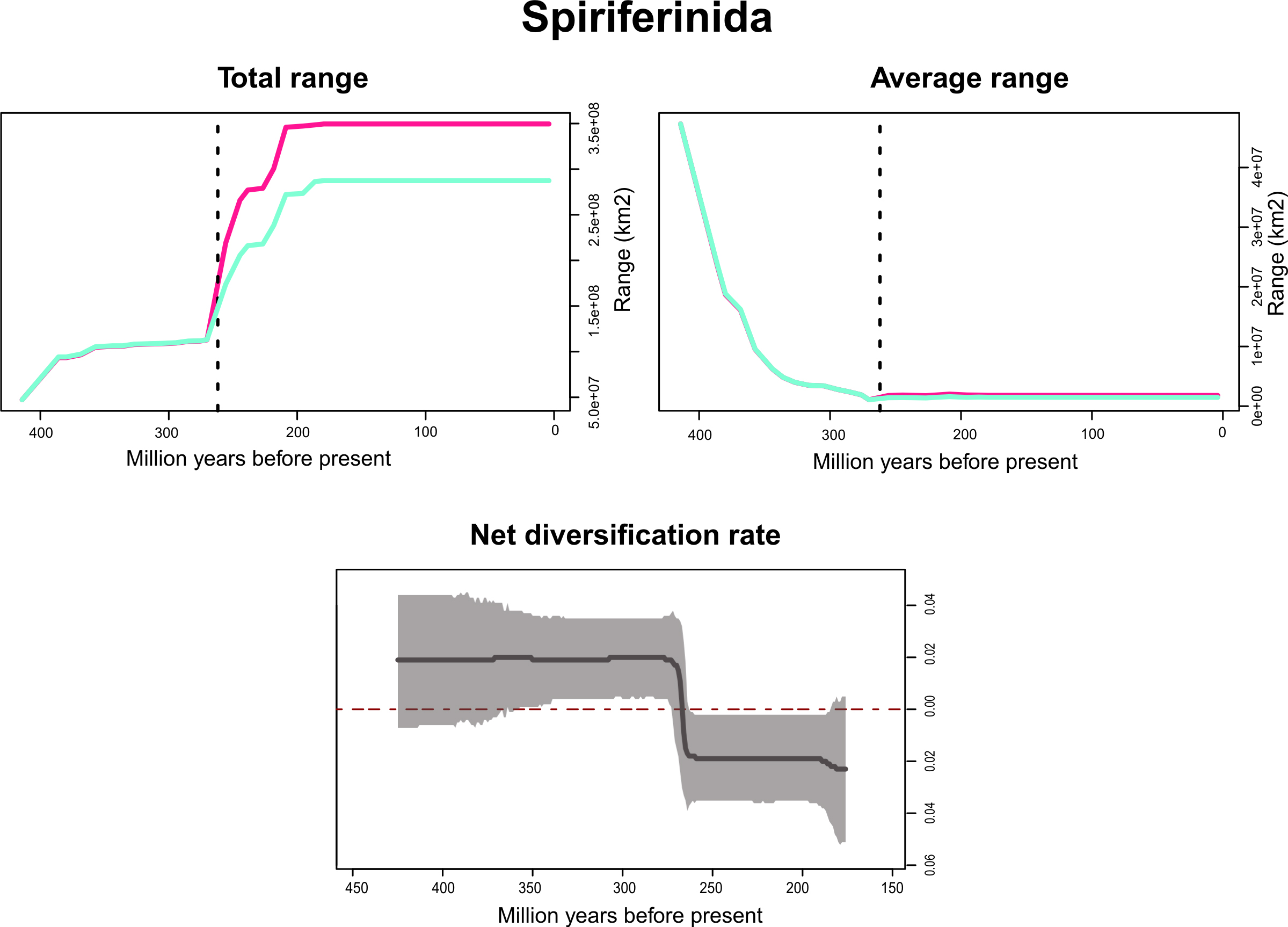

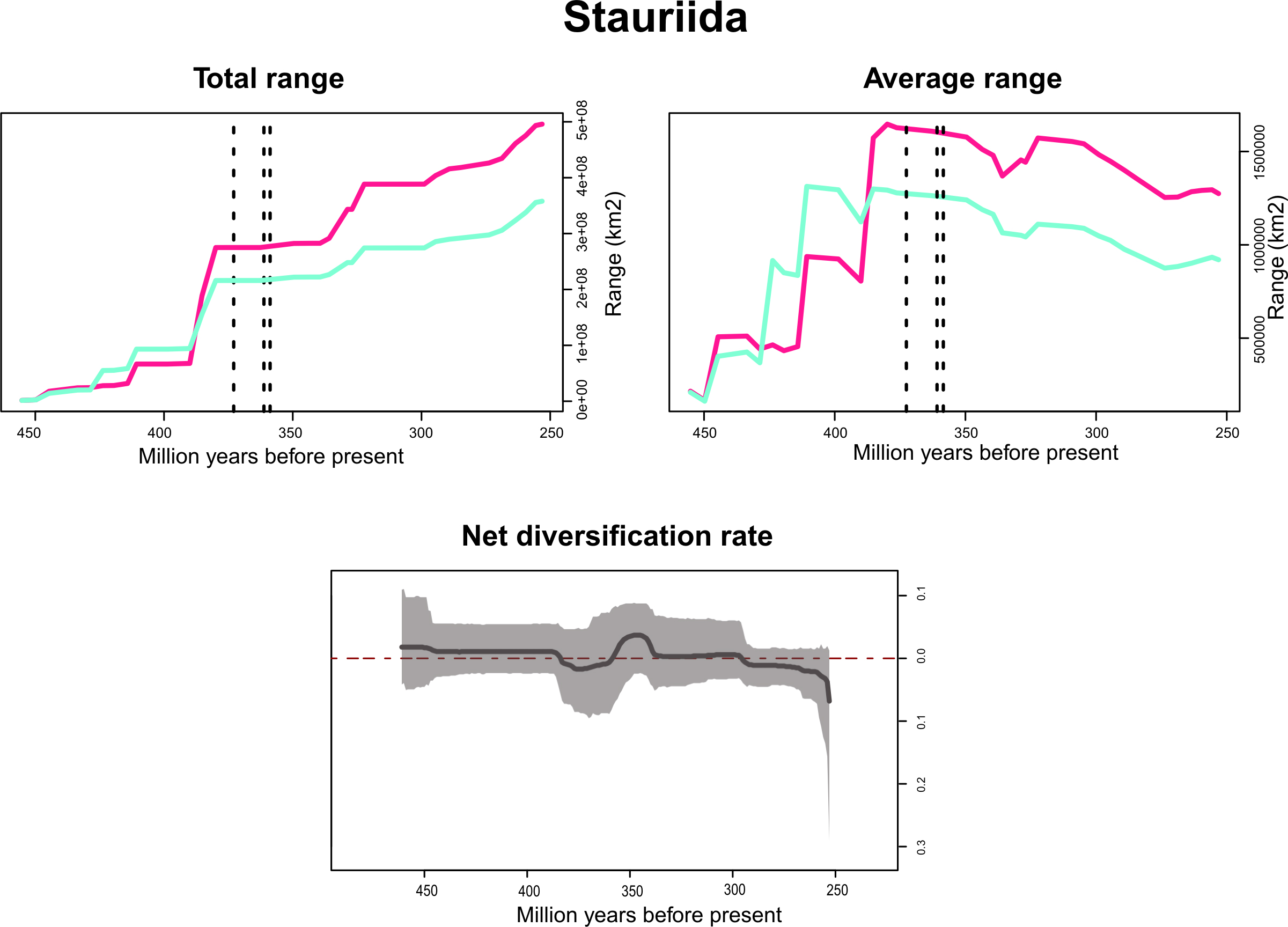

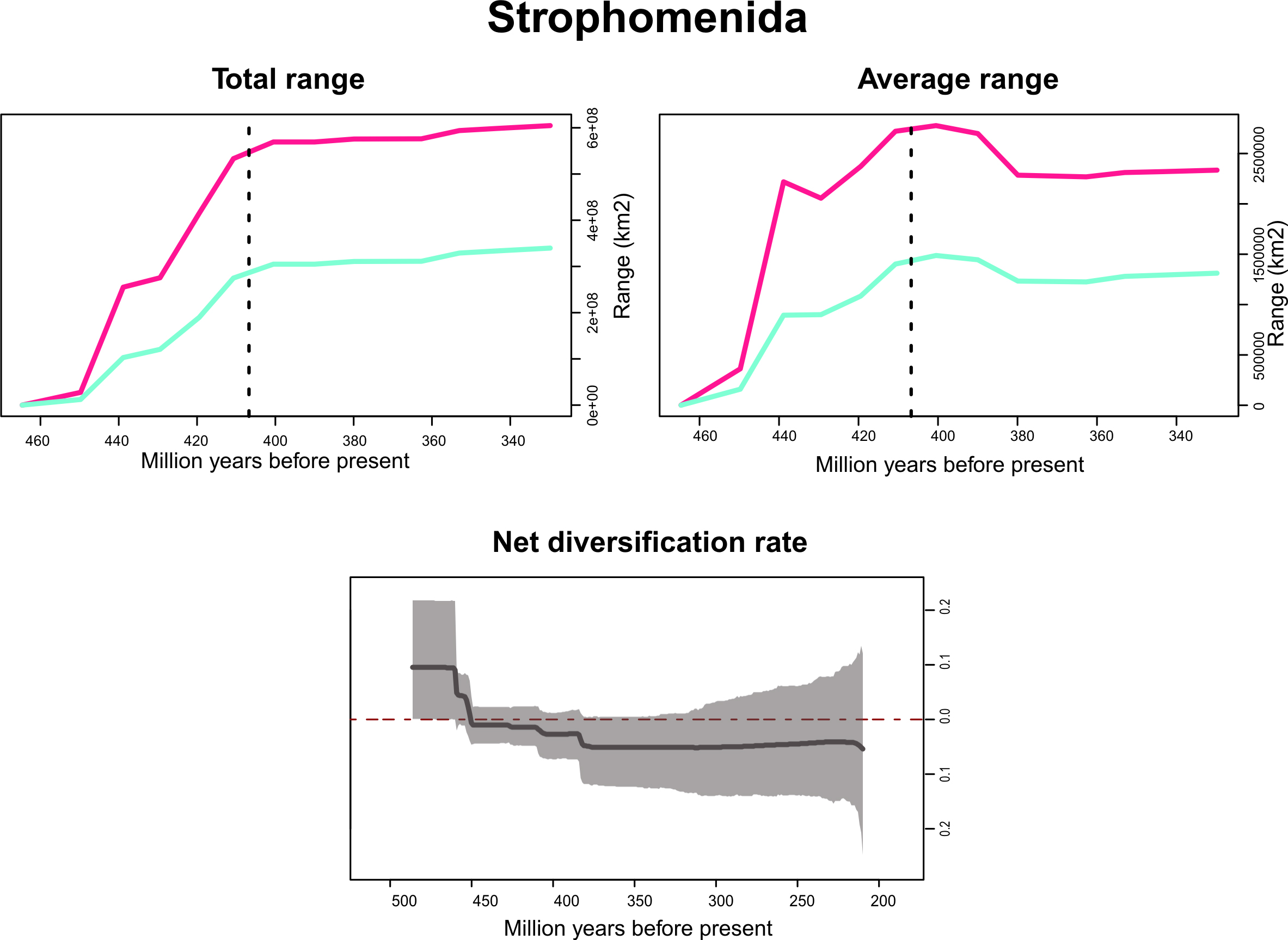

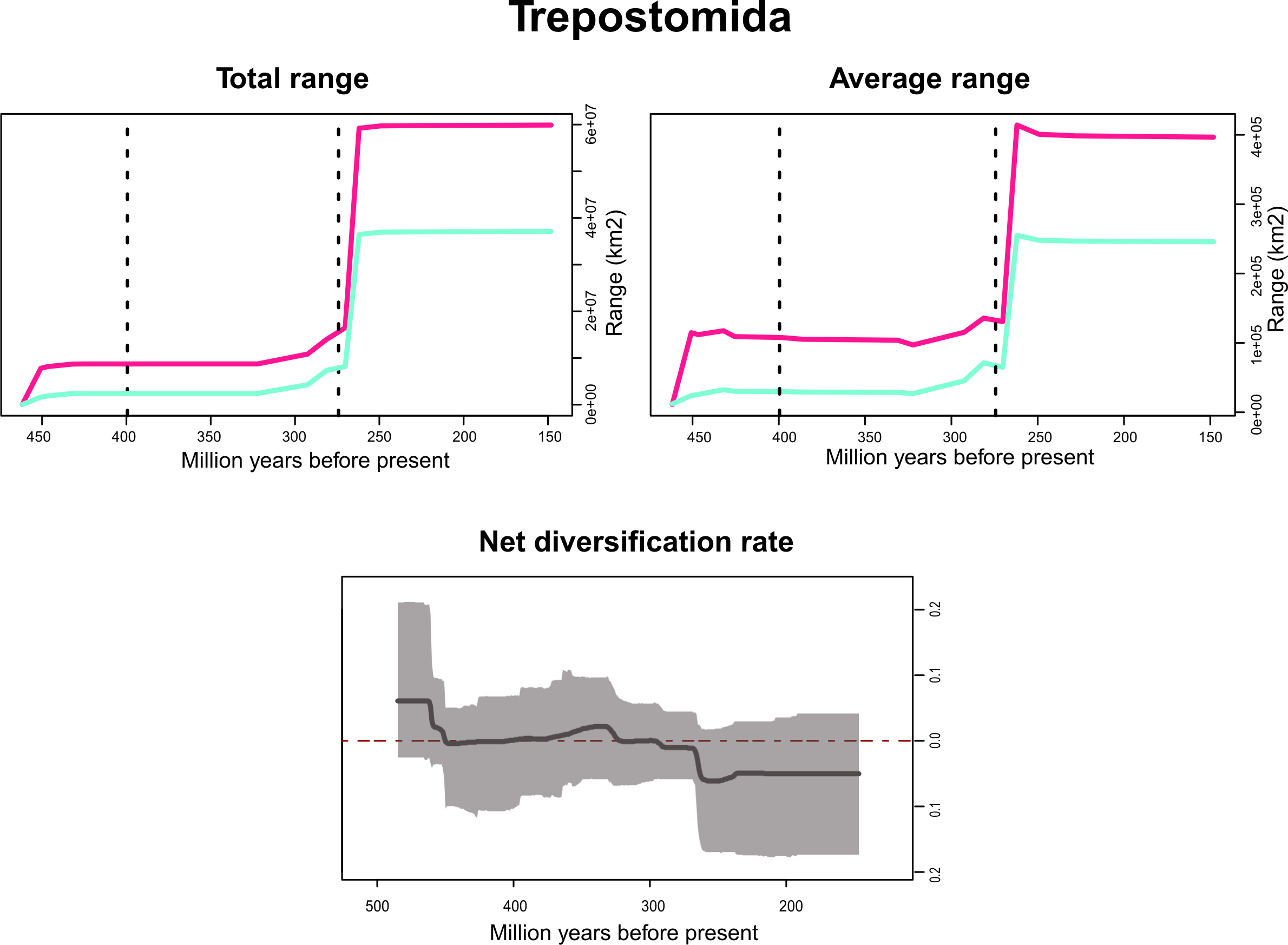
**

**Table S1. Results repeated on clades limited to species > 10 occurrences. The acronyms are the same as in table 1 in the main manuscript.**

| **clade** | **p.dist** | **shiftpoints** | **p.sym** | **p.bar** | **n.int** | **area test** | **slope** | **p** | **net.t** |
| --- | --- | --- | --- | --- | --- | --- | --- | --- | --- |
| Productida | 0.028 | 304.095 | 0.044 | 0.047 | 16.000 | 159.477 | 39755.269 | 0.069 | < 0.0001 |
| Productida | 0.000 | 363.588 | 0.298 | 0.580 | 16.000 | 126.667 | 13939.957 | 0.006 | < 0.0001 |
| Athyridida | 0.116 | 329.947 | 0.569 | 0.568 | 16.000 | 216.650 | 20621.933 | 0.006 | < 0.0001 |
| Athyridida | 0.013 | 396.669 | 0.486 | 0.998 | 16.000 | 153.930 | 8867.833 | 0.002 | < 0.0001 |
| Bellerophontidae | 0.037 | 266.396 | - | - | 4.000 | - | - | - | < 0.0001 |
| Cystoporida | 0.000 | 261.937 | - | - | 3.000 | - | - | - | < 0.0001 |
| Euomphalidae | 0.161 | 292.809 | - | - | 2.000 | - | - | - | < 0.0001 |
| Favositida | 0.025 | 417.626 | 0.032 | 0.336 | 5.000 | 51.673 | -189774.721 | 0.097 | < 0.0001 |
| Fenestrida | 0.000 | 286.741 | 0.963 | 0.206 | 6.000 | 107.084 | -20685.499 | 0.591 | < 0.0001 |
| Lophospiridae | 0.000 | 457.525 | - | - | 2.000 | - | -11779.371 | - | < 0.0001 |
| Orthida | 0.020 | 340.031 | 0.951 | 0.225 | 16.000 | 68.802 | 11451.373 | 0.043 | < 0.0001 |
| Orthida | 0.041 | 431.212 | 0.801 | 0.595 | 16.000 | 20.436 | -2174.836 | 0.334 | < 0.0001 |
| Orthotetida | 0.005 | 300.634 | 0.053 | 0.044 | 10.000 | 185.705 | 20868.774 | 0.221 | < 0.0001 |
| Pterineidae | 0.000 | 385.670 | - | - | 2.000 | - | - | - | < 0.0001 |
| Rhabdomesida | 0.000 | 287.255 | - | - | 2.000 | - | -149704.501 | - | < 0.0001 |
| Spiriferida | 0.023 | 312.587 | 0.079 | 0.171 | 18.000 | 109.946 | 2531.094 | 0.815 | < 0.0001 |
| Spiriferida | 0.000 | 383.814 | 0.168 | 0.333 | 18.000 | 97.870 | 4087.267 | 0.058 | < 0.0001 |
| Spiriferinida | 0.016 | 254.520 | 0.759 | 0.540 | 11.000 | 25.629 | -45977.747 | 0.015 | < 0.0001 |
| Stauriida | 0.031 | 305.178 | 0.161 | 0.225 | 20.000 | 9.353 | 15107.005 | 0.554 | < 0.0001 |
| Stauriida | 0.000 | 386.118 | 0.903 | 0.026 | 20.000 | 31.356 | 4115.563 | 0.455 | < 0.0001 |
| Strophomenida | 0.062 | 403.052 | 0.113 | 0.153 | 7.000 | 9.416 | 2324.737 | - | < 0.0001 |
| Trepostomida | 0.007 | 290.686 | 0.372 | 0.967 | 5.000 | 14.558 | 95064.155 | 0.095 | < 0.0001 |

**Table S2. Clade = focal clade; Bin length = duration of each bin in myr; Species per bin = number of species in each time interval. Temporal bins are collated in time, from the oldest to the youngest.**

| **Clade** | **Bin length** | **Species per bin** |
| --- | --- | --- |
| Desmoceratidae | 2 | 11 |
| Desmoceratidae | 2 | 14 |
| Desmoceratidae | 2 | 12 |
| Desmoceratidae | 2 | 3 |
| Desmoceratidae | 2 | 13 |
| Desmoceratidae | 2 | 27 |
| Desmoceratidae | 2 | 1 |
| Desmoceratidae | 2 | 25 |
| Desmoceratidae | 2 | 3 |
| Desmoceratidae | 2 | 43 |
| Desmoceratidae | 2 | 2 |
| Desmoceratidae | 2 | 28 |
| Desmoceratidae | 2 | 17 |
| Desmoceratidae | 2 | 9 |
| Desmoceratidae | 2 | 2 |
| Desmoceratidae | 2 | 7 |
| Desmoceratidae | 2 | 22 |
| Desmoceratidae | 2 | 3 |
| Desmoceratidae | 2 | 21 |
| Favositida | 5 | 2 |
| Favositida | 5 | 7 |
| Favositida | 5 | 8 |
| Favositida | 5 | 4 |
| Favositida | 5 | 11 |
| Favositida | 5 | 15 |
| Favositida | 5 | 33 |
| Favositida | 5 | 2 |
| Favositida | 5 | 8 |
| Favositida | 5 | 1 |
| Favositida | 5 | 4 |
| Favositida | 5 | 1 |
| Favositida | 5 | 6 |
| Favositida | 5 | 5 |
| Favositida | 5 | 7 |
| Favositida | 5 | 5 |
| Favositida | 5 | 5 |
| Favositida | 5 | 2 |
| Favositida | 5 | 3 |
| Favositida | 5 | 4 |
| Favositida | 5 | 9 |
| Favositida | 5 | 49 |
| Favositida | 5 | 244 |
| Favositida | 5 | 128 |
| Favositida | 5 | 26 |
| Favositida | 5 | 107 |
| Favositida | 5 | 35 |
| Favositida | 5 | 134 |
| Favositida | 5 | 80 |
| Favositida | 5 | 75 |
| Favositida | 5 | 86 |
| Favositida | 5 | 58 |
| Favositida | 5 | 59 |
| Favositida | 5 | 11 |
| Favositida | 5 | 31 |
| Favositida | 5 | 5 |
| Favositida | 5 | 3 |
| Stauriida | 5 | 99 |
| Stauriida | 5 | 67 |
| Stauriida | 5 | 105 |
| Stauriida | 5 | 68 |
| Stauriida | 5 | 143 |
| Stauriida | 5 | 16 |
| Stauriida | 5 | 109 |
| Stauriida | 5 | 86 |
| Stauriida | 5 | 100 |
| Stauriida | 5 | 41 |
| Stauriida | 5 | 28 |
| Stauriida | 5 | 41 |
| Stauriida | 5 | 18 |
| Stauriida | 5 | 63 |
| Stauriida | 5 | 15 |
| Stauriida | 5 | 104 |
| Stauriida | 5 | 81 |
| Stauriida | 5 | 68 |
| Stauriida | 5 | 41 |
| Stauriida | 5 | 63 |
| Stauriida | 5 | 11 |
| Stauriida | 5 | 17 |
| Stauriida | 5 | 4 |
| Stauriida | 5 | 13 |
| Stauriida | 5 | 59 |
| Stauriida | 5 | 225 |
| Stauriida | 5 | 331 |
| Stauriida | 5 | 108 |
| Stauriida | 5 | 37 |
| Stauriida | 5 | 62 |
| Stauriida | 5 | 54 |
| Stauriida | 5 | 89 |
| Stauriida | 5 | 80 |
| Stauriida | 5 | 49 |
| Stauriida | 5 | 97 |
| Stauriida | 5 | 85 |
| Stauriida | 5 | 55 |
| Stauriida | 5 | 9 |
| Stauriida | 5 | 77 |
| Stauriida | 5 | 36 |
| Stauriida | 5 | 19 |
| Stauriida | 5 | 1 |
| Auloporida | 5 | 1 |
| Auloporida | 5 | 7 |
| Auloporida | 5 | 13 |
| Auloporida | 5 | 23 |
| Auloporida | 5 | 2 |
| Auloporida | 5 | 4 |
| Auloporida | 5 | 23 |
| Auloporida | 5 | 7 |
| Auloporida | 5 | 1 |
| Auloporida | 5 | 5 |
| Auloporida | 5 | 2 |
| Auloporida | 5 | 3 |
| Auloporida | 5 | 8 |
| Auloporida | 5 | 6 |
| Auloporida | 5 | 7 |
| Auloporida | 5 | 6 |
| Auloporida | 5 | 3 |
| Auloporida | 5 | 8 |
| Auloporida | 5 | 3 |
| Auloporida | 5 | 1 |
| Auloporida | 5 | 4 |
| Auloporida | 5 | 10 |
| Auloporida | 5 | 41 |
| Auloporida | 5 | 26 |
| Auloporida | 5 | 2 |
| Auloporida | 5 | 3 |
| Auloporida | 5 | 2 |
| Auloporida | 5 | 10 |
| Auloporida | 5 | 3 |
| Auloporida | 5 | 15 |
| Auloporida | 5 | 12 |
| Auloporida | 5 | 10 |
| Auloporida | 5 | 12 |
| Auloporida | 5 | 2 |
| Auloporida | 5 | 1 |
| Auloporida | 5 | 10 |
| Auloporida | 5 | 8 |
| Cystiphyllida | 5 | 77 |
| Cystiphyllida | 5 | 19 |
| Cystiphyllida | 5 | 15 |
| Cystiphyllida | 5 | 20 |
| Cystiphyllida | 5 | 48 |
| Cystiphyllida | 5 | 32 |
| Cystiphyllida | 5 | 34 |
| Cystiphyllida | 5 | 37 |
| Cystiphyllida | 5 | 24 |
| Cystiphyllida | 5 | 15 |
| Cystiphyllida | 5 | 1 |
| Cystiphyllida | 5 | 1 |
| Cystiphyllida | 5 | 2 |
| Pterineidae | 7 | 4 |
| Pterineidae | 7 | 10 |
| Pterineidae | 7 | 4 |
| Pterineidae | 7 | 2 |
| Pterineidae | 7 | 1 |
| Pterineidae | 7 | 4 |
| Pterineidae | 7 | 4 |
| Pterineidae | 7 | 6 |
| Pterineidae | 7 | 10 |
| Pterineidae | 7 | 3 |
| Pterineidae | 7 | 2 |
| Pterineidae | 7 | 1 |
| Pterineidae | 7 | 7 |
| Pterineidae | 7 | 14 |
| Pterineidae | 7 | 36 |
| Pterineidae | 7 | 32 |
| Pterineidae | 7 | 5 |
| Pterineidae | 7 | 11 |
| Pterineidae | 7 | 6 |
| Pterineidae | 7 | 18 |
| Pterineidae | 7 | 20 |
| Pterineidae | 7 | 12 |
| Pterineidae | 7 | 6 |
| Pterineidae | 7 | 1 |
| Pterineidae | 7 | 6 |
| Pterineidae | 7 | 2 |
| Athyridida | 10 | 10 |
| Athyridida | 10 | 2 |
| Athyridida | 10 | 12 |
| Athyridida | 10 | 20 |
| Athyridida | 10 | 38 |
| Athyridida | 10 | 19 |
| Athyridida | 10 | 16 |
| Athyridida | 10 | 139 |
| Athyridida | 10 | 107 |
| Athyridida | 10 | 70 |
| Athyridida | 10 | 31 |
| Athyridida | 10 | 35 |
| Athyridida | 10 | 18 |
| Athyridida | 10 | 37 |
| Athyridida | 10 | 37 |
| Athyridida | 10 | 43 |
| Athyridida | 10 | 25 |
| Athyridida | 10 | 47 |
| Athyridida | 10 | 25 |
| Athyridida | 10 | 34 |
| Athyridida | 10 | 57 |
| Athyridida | 10 | 46 |
| Athyridida | 10 | 40 |
| Athyridida | 10 | 91 |
| Athyridida | 10 | 76 |
| Athyridida | 10 | 27 |
| Athyridida | 10 | 8 |
| Athyridida | 10 | 3 |
| Orthida | 10 | 49 |
| Orthida | 10 | 54 |
| Orthida | 10 | 30 |
| Orthida | 10 | 20 |
| Orthida | 10 | 37 |
| Orthida | 10 | 7 |
| Orthida | 10 | 8 |
| Orthida | 10 | 18 |
| Orthida | 10 | 15 |
| Orthida | 10 | 10 |
| Orthida | 10 | 18 |
| Orthida | 10 | 12 |
| Orthida | 10 | 30 |
| Orthida | 10 | 46 |
| Orthida | 10 | 38 |
| Orthida | 10 | 65 |
| Orthida | 10 | 109 |
| Orthida | 10 | 126 |
| Orthida | 10 | 111 |
| Orthida | 10 | 162 |
| Orthida | 10 | 265 |
| Orthida | 10 | 117 |
| Orthida | 10 | 65 |
| Orthida | 10 | 25 |
| Orthida | 10 | 6 |
| Orthida | 10 | 11 |
| Orthida | 10 | 1 |
| Orthotetida | 10 | 122 |
| Orthotetida | 10 | 93 |
| Orthotetida | 10 | 63 |
| Orthotetida | 10 | 62 |
| Orthotetida | 10 | 52 |
| Orthotetida | 10 | 10 |
| Orthotetida | 10 | 14 |
| Orthotetida | 10 | 11 |
| Orthotetida | 10 | 14 |
| Orthotetida | 10 | 10 |
| Orthotetida | 10 | 14 |
| Orthotetida | 10 | 12 |
| Orthotetida | 10 | 20 |
| Orthotetida | 10 | 18 |
| Orthotetida | 10 | 8 |
| Orthotetida | 10 | 14 |
| Orthotetida | 10 | 40 |
| Orthotetida | 10 | 44 |
| Orthotetida | 10 | 14 |
| Orthotetida | 10 | 26 |
| Orthotetida | 10 | 21 |
| Orthotetida | 10 | 3 |
| Productida | 10 | 403 |
| Productida | 10 | 672 |
| Productida | 10 | 424 |
| Productida | 10 | 290 |
| Productida | 10 | 347 |
| Productida | 10 | 93 |
| Productida | 10 | 83 |
| Productida | 10 | 164 |
| Productida | 10 | 153 |
| Productida | 10 | 85 |
| Productida | 10 | 114 |
| Productida | 10 | 47 |
| Productida | 10 | 51 |
| Productida | 10 | 87 |
| Productida | 10 | 35 |
| Productida | 10 | 56 |
| Productida | 10 | 35 |
| Productida | 10 | 22 |
| Productida | 10 | 6 |
| Productida | 10 | 3 |
| Productida | 10 | 1 |
| Spiriferida | 10 | 138 |
| Spiriferida | 10 | 166 |
| Spiriferida | 10 | 199 |
| Spiriferida | 10 | 137 |
| Spiriferida | 10 | 163 |
| Spiriferida | 10 | 54 |
| Spiriferida | 10 | 71 |
| Spiriferida | 10 | 26 |
| Spiriferida | 10 | 88 |
| Spiriferida | 10 | 75 |
| Spiriferida | 10 | 106 |
| Spiriferida | 10 | 76 |
| Spiriferida | 10 | 60 |
| Spiriferida | 10 | 253 |
| Spiriferida | 10 | 122 |
| Spiriferida | 10 | 120 |
| Spiriferida | 10 | 176 |
| Spiriferida | 10 | 131 |
| Spiriferida | 10 | 39 |
| Spiriferida | 10 | 5 |
| Spiriferinida | 10 | 41 |
| Spiriferinida | 10 | 39 |
| Spiriferinida | 10 | 25 |
| Spiriferinida | 10 | 42 |
| Spiriferinida | 10 | 34 |
| Spiriferinida | 10 | 21 |
| Spiriferinida | 10 | 46 |
| Spiriferinida | 10 | 29 |
| Spiriferinida | 10 | 76 |
| Spiriferinida | 10 | 77 |
| Spiriferinida | 10 | 32 |
| Spiriferinida | 10 | 35 |
| Spiriferinida | 10 | 10 |
| Spiriferinida | 10 | 15 |
| Spiriferinida | 10 | 10 |
| Spiriferinida | 10 | 14 |
| Spiriferinida | 10 | 25 |
| Spiriferinida | 10 | 31 |
| Spiriferinida | 10 | 8 |
| Spiriferinida | 10 | 2 |
| Spiriferinida | 10 | 17 |
| Spiriferinida | 10 | 6 |
| Spiriferinida | 10 | 8 |
| Spiriferinida | 10 | 7 |
| Spiriferinida | 10 | 2 |
| Strophomenida | 10 | 1 |
| Strophomenida | 10 | 2 |
| Strophomenida | 10 | 2 |
| Strophomenida | 10 | 3 |
| Strophomenida | 10 | 2 |
| Strophomenida | 10 | 4 |
| Strophomenida | 10 | 115 |
| Strophomenida | 10 | 40 |
| Strophomenida | 10 | 84 |
| Strophomenida | 10 | 133 |
| Strophomenida | 10 | 135 |
| Strophomenida | 10 | 128 |
| Strophomenida | 10 | 92 |
| Strophomenida | 10 | 313 |
| Strophomenida | 10 | 64 |
| Strophomenida | 10 | 8 |
| Strophomenida | 10 | 1 |
| Cystoporida | 10 | 1 |
| Cystoporida | 10 | 12 |
| Cystoporida | 10 | 49 |
| Cystoporida | 10 | 50 |
| Cystoporida | 10 | 10 |
| Cystoporida | 10 | 52 |
| Cystoporida | 10 | 14 |
| Cystoporida | 10 | 11 |
| Cystoporida | 10 | 16 |
| Cystoporida | 10 | 5 |
| Cystoporida | 10 | 16 |
| Cystoporida | 10 | 5 |
| Cystoporida | 10 | 22 |
| Cystoporida | 10 | 5 |
| Cystoporida | 10 | 6 |
| Cystoporida | 10 | 24 |
| Cystoporida | 10 | 5 |
| Cystoporida | 10 | 1 |
| Fenestrida | 10 | 1 |
| Fenestrida | 10 | 59 |
| Fenestrida | 10 | 98 |
| Fenestrida | 10 | 148 |
| Fenestrida | 10 | 126 |
| Fenestrida | 10 | 92 |
| Fenestrida | 10 | 38 |
| Fenestrida | 10 | 24 |
| Fenestrida | 10 | 39 |
| Fenestrida | 10 | 35 |
| Fenestrida | 10 | 28 |
| Fenestrida | 10 | 3 |
| Fenestrida | 10 | 2 |
| Fenestrida | 10 | 1 |
| Fenestrida | 10 | 17 |
| Fenestrida | 10 | 7 |
| Fenestrida | 10 | 7 |
| Fenestrida | 10 | 7 |
| Fenestrida | 10 | 5 |
| Fenestrida | 10 | 4 |
| Rhabdomesida | 10 | 1 |
| Rhabdomesida | 10 | 1 |
| Rhabdomesida | 10 | 6 |
| Rhabdomesida | 10 | 24 |
| Rhabdomesida | 10 | 41 |
| Rhabdomesida | 10 | 14 |
| Rhabdomesida | 10 | 46 |
| Rhabdomesida | 10 | 16 |
| Rhabdomesida | 10 | 8 |
| Rhabdomesida | 10 | 10 |
| Rhabdomesida | 10 | 5 |
| Rhabdomesida | 10 | 12 |
| Rhabdomesida | 10 | 2 |
| Rhabdomesida | 10 | 2 |
| Rhabdomesida | 10 | 2 |
| Rhabdomesida | 10 | 19 |
| Rhabdomesida | 10 | 6 |
| Rhabdomesida | 10 | 4 |
| Rhabdomesida | 10 | 4 |
| Rhabdomesida | 10 | 5 |
| Rhabdomesida | 10 | 4 |
| Rhabdomesida | 10 | 16 |
| Rhabdomesida | 10 | 6 |
| Trepostomida | 10 | 1 |
| Trepostomida | 10 | 1 |
| Trepostomida | 10 | 2 |
| Trepostomida | 10 | 3 |
| Trepostomida | 10 | 2 |
| Trepostomida | 10 | 9 |
| Trepostomida | 10 | 3 |
| Trepostomida | 10 | 39 |
| Trepostomida | 10 | 70 |
| Trepostomida | 10 | 76 |
| Trepostomida | 10 | 41 |
| Trepostomida | 10 | 28 |
| Trepostomida | 10 | 7 |
| Trepostomida | 10 | 6 |
| Trepostomida | 10 | 10 |
| Trepostomida | 10 | 13 |
| Trepostomida | 10 | 5 |
| Trepostomida | 10 | 3 |
| Trepostomida | 10 | 3 |
| Trepostomida | 10 | 26 |
| Trepostomida | 10 | 12 |
| Trepostomida | 10 | 13 |
| Trepostomida | 10 | 51 |
| Trepostomida | 10 | 36 |
| Trepostomida | 10 | 24 |
| Trepostomida | 10 | 69 |
| Trepostomida | 10 | 251 |
| Trepostomida | 10 | 28 |
| Trepostomida | 10 | 4 |
| Trepostomida | 10 | 2 |
| Bellerophontidae | 20 | 1 |
| Bellerophontidae | 20 | 28 |
| Bellerophontidae | 20 | 30 |
| Bellerophontidae | 20 | 10 |
| Bellerophontidae | 20 | 21 |
| Bellerophontidae | 20 | 38 |
| Bellerophontidae | 20 | 19 |
| Bellerophontidae | 20 | 7 |
| Bellerophontidae | 20 | 25 |
| Bellerophontidae | 20 | 14 |
| Bellerophontidae | 20 | 18 |
| Bellerophontidae | 20 | 21 |
| Bellerophontidae | 20 | 5 |
| Euomphalidae | 20 | 4 |
| Euomphalidae | 20 | 1 |
| Euomphalidae | 20 | 4 |
| Euomphalidae | 20 | 1 |
| Euomphalidae | 20 | 4 |
| Euomphalidae | 20 | 1 |
| Euomphalidae | 20 | 4 |
| Euomphalidae | 20 | 12 |
| Euomphalidae | 20 | 15 |
| Euomphalidae | 20 | 18 |
| Euomphalidae | 20 | 15 |
| Euomphalidae | 20 | 28 |
| Euomphalidae | 20 | 11 |
| Euomphalidae | 20 | 23 |
| Euomphalidae | 20 | 16 |
| Euomphalidae | 20 | 32 |
| Euomphalidae | 20 | 11 |
| Euomphalidae | 20 | 3 |
| Euomphalidae | 20 | 2 |
| Lophospiridae | 20 | 2 |
| Lophospiridae | 20 | 1 |
| Lophospiridae | 20 | 5 |
| Lophospiridae | 20 | 29 |
| Lophospiridae | 20 | 24 |
| Lophospiridae | 20 | 17 |
| Lophospiridae | 20 | 9 |
| Lophospiridae | 20 | 4 |
| Lophospiridae | 20 | 3 |
| Lophospiridae | 20 | 1 |
| Lophospiridae | 20 | 2 |
| Lophospiridae | 20 | 2 |
| Lophospiridae | 20 | 12 |
| Lophospiridae | 20 | 32 |
| Lophospiridae | 20 | 43 |
| Lophospiridae | 20 | 5 |
| Proetidae | 10 | 1 |
| Proetidae | 10 | 2 |
| Proetidae | 10 | 4 |
| Proetidae | 10 | 8 |
| Proetidae | 10 | 24 |
| Proetidae | 10 | 24 |
| Proetidae | 10 | 138 |
| Proetidae | 10 | 18 |
| Proetidae | 10 | 5 |
| Proetidae | 10 | 61 |
| Proetidae | 10 | 23 |
| Proetidae | 10 | 21 |
| Proetidae | 10 | 95 |
| Proetidae | 10 | 39 |
| Proetidae | 10 | 17 |
| Proetidae | 10 | 10 |
| Proetidae | 10 | 16 |
| Proetidae | 10 | 2 |

**R script used to compute range size evolution in clades**

The object “aulas” contains the record of a single clade, the object “Aulo.rates” includes the diversification statistics per million year. “aulas” file looks as this:

| Major | Clade | Midage | RangeInt | cum_spec | cum_clade | num_spec | spec_area | clade_area | sympatry |
| --- | --- | --- | --- | --- | --- | --- | --- | --- | --- |
| Trilobita | Tropidocoryphidae | 420 | 5 | 41137.69 | 21259 | 4 | 41137.69 | 21259 | 0.4832232 |
| Trilobita | Tropidocoryphidae | 410.7 | 4 | 55806.11 | 32842.4 | 4 | 14668.43 | 11583.4 | 0.411491 |
| Trilobita | Tropidocoryphidae | 398 | 3 | 74079.76 | 40430.07 | 4 | 18273.65 | 7587.67 | 0.454236 |
| Trilobita | Tropidocoryphidae | 390 | 2 | 607941.82 | 478438.07 | 9 | 533862.06 | 438008 | 0.21302 |

**Cum_spec represents the sum of individual range size, cum_clade the clade level range size. All of these data must be prepared in advance in a GIS implementation.**

The object “Aulo.rates” is produced by PyRate software, it looks this way:

|  | time.Ma. | sp_rate | ex_rate | net_div |
| --- | --- | --- | --- | --- |
| 380 | 379-380 | 0.021 | 0.865 | -0.844 |
| 381 | 380-381 | 0.021 | 0.865 | -0.844 |
| 382 | 381-382 | 0.021 | 0.865 | -0.844 |
| 383 | 382-383 | 0.021 | 0.865 | -0.844 |
| 384 | 383-384 | 0.021 | 0.865 | -0.844 |
| 385 | 384-385 | 0.021 | 0.865 | -0.844 |

**goldenpoints<-function(aulas,Aulo.rates)**

**{**

**library(breakpoint)**

**library(lawstat)**

**### compute the shifts first ###**

**dim(aulas)[1]->n.int**

**if(inherits(try(aulas[CE.Normal(as.data.frame(cumsum(aulas$sym)))$BP,]$Mid,silent=T),"try-error")==TRUE) sym.age<-aulas[which.max(diff(cumsum(aulas$sym)))+1,]$Mid else sym.age<-aulas[CE.Normal(as.data.frame(cumsum(aulas$sym)))$BP,]$Mid**

**if(inherits(try(aulas[CE.Normal(as.data.frame(aulas$cum_c))$BP,]$Mid,silent=T),"try-error")==TRUE) range.age<-aulas[which.max(diff(aulas$cum_c))+1,]$Mid else range.age<-aulas[CE.Normal(as.data.frame(aulas$cum_c))$BP,]$Mid**

**if(inherits(try(aulas[CE.Normal(as.data.frame(aulas$cum_sp))$BP,]$Mid,silent=T),"try-error")==TRUE) cumspec.age<-aulas[which.max(diff(aulas$cum_sp))+1,]$Mid else cumspec.age<-aulas[CE.Normal(as.data.frame(aulas$cum_sp))$BP,]$Mid**

**if(inherits(try(aulas[CE.Normal(as.data.frame(aulas$spec_a))$BP,]$Mid,silent=T),"try-error")==TRUE) specarea.age<-aulas[which.max(diff(aulas$spec_a))+1,]$Mid else specarea.age<-aulas[CE.Normal(as.data.frame(aulas$spec_a))$BP,]$Mid**

**if(inherits(try(aulas[CE.Normal(as.data.frame(aulas$num))$BP,]$Mid,silent=T),"try-error")==TRUE) rich.age<-aulas[which.max(diff(aulas$num))+1,]$Mid else rich.age<-aulas[CE.Normal(as.data.frame(aulas$num))$BP,]$Mid**

**if(inherits(try(rownames(Aulo.rates[CE.Normal(as.data.frame(Aulo.rates[,4]))$BP,]),silent=T),"try-error")==TRUE) net.age<-rownames(Aulo.rates[which.max(diff(Aulo.rates[,4])),]) else rownames(Aulo.rates[CE.Normal(as.data.frame(Aulo.rates[,4]))$BP,])->net.age**

**min(c(length(net.age),length(range.age),length(sym.age)))->n.breaks**

**c("net","range","sym")->vars**

**vars[which((c(length(net.age),length(range.age),length(sym.age)))>n.breaks)]->multimodel**

**if(length(multimodel>0))**

**{**

**vars[-which(vars%in%multimodel)]->break.model**

**data.frame(c(net.age,sym.age,range.age),c(rep("net",length(net.age)),rep("sym",length(sym.age)),rep("range",length(range.age))))->ages**

**names(ages)<-c("age","var")**

**ages[ages[,2]%in%multimodel,]->choose**

**ages[ages[,2]!=multimodel,]->stay**

**break.tar<-list()**

**for(i in 1:n.breaks){**

**stays<-list()**

**for(j in 1:length(unique(stay[,2]))){**

**stay[stay[,2]==unique(stay[,2])[j],][i,]->stays[[j]]**

**}**

**do.call(rbind,stays)->break.tar[[i]]**

**}**

**target<-list()**

**for(i in 1:n.breaks){**

**as.numeric(as.character(break.tar[[i]][,1]))->target[[i]]**

**}**

**theys<-list()**

**for(z in 1:length(target)){**

**they<-list()**

**for(j in 1:length(target[[z]])){**

**you<-array()**

**for(i in 1:dim(choose)[1]){**

**abs(as.numeric(as.character(choose[,1][i])) - target[[z]][j])->you[i]**

**}**

**data.frame(you, choose[,2])->they[[j]]**

**}**

**list(they)->theys[[z]]**

**}**

**to.stays<-list()**

**for(i in 1:n.breaks){**

**choose[which.min(apply(t(matrix(unlist(theys[i]),ncol=2)),2,sum)[1:dim(choose)[1]]),]->to.stays[[i]]**

**}**

**new.age<-list()**

**for(i in 1:n.breaks){**

**rbind(break.tar[[i]],to.stays[[i]])->new.age[[i]]**

**}**

**sum.dist<-array()**

**for(i in 1:length(new.age)){**

**sum(dist(new.age[[i]][,1]))->sum.dist[i]**

**}**

**} else {**

**sum(dist(ages[,1]))->sum.dist**

**}**

**###### once the sum of distance between shift points is estimated, it could be tested against random distances to assess significance in shift points temporal coincidence #####**

**ran.dist<-array()**

**for(i in 1:9999){**

**sum(dist(c(sample(aulas$Mid,2),sample(rownames(Aulo.rates),1))))->ran.dist[i]**

**}**

**p.dist<-array()**

**for(i in 1:n.breaks){**

**length(which(ran.dist<=sum.dist[i]))/length(ran.dist)->p.dist[i]**

**}**

**breakpoint<-array()**

**for(i in 1:n.breaks){**

**mean(as.numeric(as.character(new.age[[i]][,1])))->breakpoint[i]**

**}**

**sym.part<-list()**

**for(i in 1:n.breaks){**

**data.frame(aulas$sym,c(rep("before",dim(aulas[aulas$Mid>breakpoint[i],])[1]),rep("after",dim(aulas[aulas$Mid<breakpoint[i],])[1])))->sym.part[[i]]**

**names(sym.part[[i]])[2]<-"when"**

**}**

**p.sym.test<-array()**

**try(for(i in 1:n.breaks){**

**lnested.test(sym.part[[i]]$aul,sym.part[[i]]$whe, tail="right",correction.method="correction.factor")$L$p.value->p.sym.test[i]**

**},silent=T)**

**p.bart<-array()**

**try(for(i in 1:n.breaks){**

**bartlett.test(sym.part[[i]][,1],sym.part[[i]][,2])[3]->p.bart[i]**

**},silent=T)**

**res<-list(new.age,breakpoint, p.dist, p.sym.test,p.bart,cumspec.age,specarea.age,rich.age,n.int)**

**names(res)<-c("new.age","breakpoint","p.dist","p.sym.test","p.bart","cumspec.age","specarea.age","rich.age","n.int")**

**return(res)**

**}**

**### end of goldenpoints ###**

**na.omit(match(unique(clade.results$Clade),names(wtf)))->order**

**wtf[order]->wtf2**

**as.character(unique(clade.results$Clade)[match(names(wtf2),unique(clade.results$Clade))])->fuck**

**clade.results[which(clade.results$Clade%in%fuck,T),]->clade.results2**

**hereUgo<-list()**

**for(i in 1:length(wtf2)){**

**###for(i in 1:10){**

**wtf2[[i]]->Aulo.rates**

**clade.results2[clade.results2$Clade==unique(clade.results2$Clade)[i],]->aulas**

**try(goldenpoints(aulas,Aulo.rates))->hereUgo[[i]]**

**names(hereUgo)[i]<-names(wtf2)[i]**

**}**

**dist.probs<-list()**

**for(i in 1:199){**

**unlist(hereUgo[[i]]$p.dist)->dist.probs[[i]]**

**data.frame(rep(names(wtf2)[i]),dist.probs[[i]])->dist.probs[[i]]**

**data.frame(dist.probs[[i]],unlist(hereUgo[[i]]$breakpo),unlist(hereUgo[[i]]$p.sym),unlist(hereUgo[[i]]$p.bar))->dist.probs[[i]]**

**data.frame(dist.probs[[i]],rep(unlist(hereUgo[[i]]$n.int),dim(dist.probs[[i]])[1]))->dist.probs[[i]]**

**names(dist.probs[[i]])<-c("clade","p.dist","breakpoints","p.sym","p.bar","n.int")**

**}**

**do.call(rbind,dist.probs)->summary**

**##### AREA TEST ###**

**area.btw<-function (x, f1, f2, xrange = c(0, 1))**

**{**

**a <- 0**

**for (i in 1:length(x)) {**

**if (x[i] >= xrange[1] & x[i] <= xrange[2]) {**

**if (i == 1) {**

**lhs <- 0**

**}**

**else if (x[i - 1] < xrange[1]) {**

**lhs <- xrange[1]**

**}**

**else lhs <- x[i - 1]**

**if (i == length(x)) {**

**rhs <- x[i]**

**}**

**else if (x[i + 1] > xrange[2]) {**

**rhs <- xrange[2]**

**}**

**else rhs <- x[i + 1]**

**a <- a + (f2[i] - f1[i]) * (rhs - lhs)/2**

**}**

**else if (i != 1)**

**if (x[i - 1] >= xrange[1] & x[i - 1] <= xrange[2]) {**

**y1 <- f1[i - 1] + (f1[i] - f1[i - 1]) * (xrange[2] -**

**x[i - 1])/(x[i] - x[i - 1])**

**y2 <- f2[i - 1] + (f2[i] - f2[i - 1]) * (xrange[2] -**

**x[i - 1])/(x[i] - x[i - 1])**

**a <- a + (y2 - y1) * (xrange[2] - x[i - 1])/2**

**}**

**else if (i != length(x))**

**if (x[i + 1] >= xrange[1] & x[i + 1] <= xrange[2]) {**

**y1 <- f1[i] + (f1[i + 1] - f1[i]) * (xrange[1] -**

**x[i])/(x[i + 1] - x[i])**

**y2 <- f2[i] + (f2[i + 1] - f2[i]) * (xrange[1] -**

**x[i])/(x[i + 1] - x[i])**

**a <- a + (y2 - y1) * (x[i + 1] - xrange[1])/2**

**}**

**}**

**return(a)**

**}**

**area.test<-list()**

**as.character(unique(summary$clade))->clas**

**###for(i in 1:dim(summary)[1]){**

**for(i in 1:length(clas)){**

**max(clade.results2[clade.results2$Clade==clas[i],]$Mid)->start**

**min(clade.results2[clade.results2$Clade==clas[i],]$Mid)->end**

**summary[summary$clade==clas[i],]$bre->breaks**

**na.omit(breaks)->breaks**

**area.diff<-array()**

**for(j in 1:length(breaks)){**

**area.btw(rev(clade.results2[clade.results2$Clade==clas[i],]$Mid),clade.results2[clade.results2$Clade==clas[i],]$cum_c,clade.results2[clade.results2$Clade==clas[i],]$cum_s,xrange=c(end,breaks[j]))->area1**

**area.btw(rev(clade.results2[clade.results2$Clade==clas[i],]$Mid),clade.results2[clade.results2$Clade==clas[i],]$cum_c,clade.results2[clade.results2$Clade==clas[i],]$cum_s,xrange=c(breaks[j], start))->area2**

**(area1/(breaks[j]-end))/(area2/(start-breaks[j]))*100->area.diff[j]**

**}**

**area.diff->area.test[[i]]**

**}**

**summary[-which(is.na(summary$breakpoints)),]->summaryX**

**data.frame(summaryX,unlist(area.test))->summary**

**### SLOPE TEST ###**

**as.character(unique(summary$clade))->cla**

**p.ran<-list()**

**for(i in 1:length(cla)){**

**clade.results2[clade.results2$Clade==cla[i],]$Mid->time**

**cumsum(clade.results2[clade.results2$Clade==cla[i],]$spec)/cumsum(clade.results2[clade.results2$Clade==cla[i],]$num)->av.range**

**summary[summary$clade==cla[i],]$bre->breaks**

**na.omit(breaks)->breaks**

**data.frame(time,av.range)->range.course**

**range<-list()**

**when<-matrix(ncol=length(breaks),nrow=dim(range.course)[1])**

**for(z in 1:dim(range.course)[1]){**

**for(j in 1:length(breaks)){**

**if(range.course$time[z]>breaks[j]) when[z,j]<-"before" else when[z,j]<-"after"**

**}**

**}**

**data.frame(range.course,when)->range**

**regr.stat<-matrix(ncol=2,nrow=length(breaks))**

**for(w in 1:length(breaks)){**

**try(summary(lm(range[range[,2+w]=="after",]$av~rev(range[range[,2+w]=="after",]$tim)))$coeff[2,c(1,4)]->regr.stat[w,],silent=T)**

**}**

**regr.stat->p.ran[[i]]**

**}**

**do.call(rbind,p.ran)->p.ran**

**colnames(p.ran)<-c("slope","p")**

**data.frame(summary,p.ran)->summary3**

**### limiting to large clades only**

**summary3[summary3$n.int>10,]->summary4**

**###NET DIVERSIFICATION RATE TEST ###**

**net.t<-array()**

**for(i in 1:dim(summary4)[1]){**

**summary4[i,]$bre->breaks**

**summary4[i,]$cla->iss**

**wtf2[[which(names(wtf2)==as.character(iss))]][,4]->net.rate**

**data.frame(do.call(rbind,strsplit(as.character(wtf2[[which(names(wtf2)==as.character(iss))]][,1]),"-"))[,2])->time**

**names(time)<-"time"**

**data.frame(time,net.rate)->netted**

**as.numeric(as.character(netted$time))->netted$time**

**wen<-array()**

**for(z in 1:dim(netted)[1]){**

**if(netted$time[z]>breaks) wen[z]<-"before" else wen[z]<-"after"**

**}**

**data.frame(netted,wen)->timed**

**try(wilcox.test(timed[timed$wen=="after",]$net,timed[timed$wen=="before",]$net,alternative="less")$p.value->net.t[i],silent=T)**

**}**

**data.frame(summary4,net.t)->summary4**

**##################**

**### net rate random test ###**

**tapply(summary4$breakp,as.character(summary4$clade),length)->real.breaks**

**reps<-999**

**hits<-array()**

**for(j in 1:reps){**

**net.ran<-list()**

**as.character(unique(summary4$cla))->cla4**

**for(i in 1:length(cla4)){**

**wtf2[[which(names(wtf2)==cla4[i])]][,4]->net.rate**

**data.frame(do.call(rbind,strsplit(as.character(wtf2[[which(names(wtf2)==cla4[i])]][,1]),"-"))[,2])->time**

**names(time)<-"time"**

**data.frame(time,net.rate)->netted**

**as.numeric(as.character(netted$time))->netted$time**

**real.breaks[match(cla4[i],names(real.breaks))]->breaks**

**max(summary4[summary4$cla==names(breaks),]$breakp)->top.age**

**names(breaks)<-NULL**

**sample(netted[,1][netted[,1]>top.age],breaks)->breaks**

**wat<-matrix(nrow=dim(netted)[1],ncol=length(breaks))**

**for(w in 1:length(breaks)){**

**wen<-array()**

**for(z in 1:dim(netted)[1]){**

**if(netted$time[z]>breaks[w]) wen[z]<-"before" else wen[z]<-"after"**

**}**

**wen->wat[,w]**

**}**

**data.frame(netted,wat)->timed**

**thats<-array()**

**for (a in 1:length(breaks)){**

**try(wilcox.test(timed[timed[,2+a]=="after",]$net,timed[timed[,2+a]=="before",]$net,alternative="less")$p.value->thats[a],silent=T)**

**}**

**thats->net.ran[[i]]**

**}**

**length(which(unlist(net.ran)<0.05))->hits[j]**

**}**

**length(which(hits>28))/(reps+1)->p.net.ran**

**#####################**

**### searching for saturation in total range curve, all clades at once ####**

**sat<-matrix(ncol=3,nrow=length(cla4))**

**for(i in 1:length(cla4)){**

**try(lm(clade.results2[clade.results2$Cla==cla4[i],]$cum_c~clade.results2[clade.results2$Cla==cla4[i],]$Mid)->lm.fit,silent=T)**

**try(logLik(lm.fit)->LL.fit,silent=T)**

**clade.results2[clade.results2$Cla==cla4[i],]$cum_c->y**

**rev(clade.results2[clade.results2$Cla==cla4[i],]$Mid)->x**

**sigmo<-function(a)**

**{**

**x-mean(x)->t**

**sum(abs((1/((2.718^-t)+1)*a)-y))**

**}**

**try(mle(sigmo,start=list(a=max(y)/2),method = "L-BFGS-B", lower= max(y))->fit.sigm,silent=T)**

**try(logLik(fit.sigm)->LL.sigm,silent=T)**

**richard<-function(b,v,q)**

**{**

**K=max(y)**

**x-mean(x)->t**

**A=min(y)**

**sum(abs(A+((K-A)/(1+q*2.718^-(b*t))^(1/v))-y))**

**}**

**try(mle(richard,start=list(b=1,v=1,q=min(y)/100),method = "L-BFGS-B",lower=c(.1,.5,100))->fit.rich,silent=T)**

**try(logLik(fit.rich)->LL.rich,silent=T)**

**c(LL.fit,LL.sigm,LL.rich)->sat[i,]**

**}**

**colnames(sat)<-c("linear","sigmoid","richard")**

**rownames(sat)<-cla4**

**### object sat could be passed to likelihood ratio test or any other alternative to compare AICc ##**

**###########**

**################**

**### SUMMARY STATISTICS ####**

**###distance equality**

**## how many breaks are significant**

**length(which(summary4$p.dist<0.06))/dim(summary4)[1]x**

**#their p**

**binom.test(length(which(summary4$p.dist<0.06)),dim(summary4)[1],0.5)$p.value**

**### how many groups show the clustering of breakpoints**

**length(which(tapply(summary4$p.dist,as.character(summary4$clade),min)<0.06))/length(unique(summary4$clade))**

**binom.test(length(which(tapply(summary4$p.dist,as.character(summary4$clade),min)<0.06)),length(unique(summary4$clade)),0.5)$p.value**

**### area test**

**##how many**

**length(which(summary4$unlist.area.test<100))/dim(summary4)[1]**

**binom.test(length(which(summary4$unlist.area.test<100)),dim(summary4)[1])$p.value**

**### slope test**

**## how many negative slopes**

**# how many negative and significant slopes**

**length(which(summary4$slope<0 & summary4$p <0.05))/length(which(summary4$p<0.05))**

**binom.test(length(which(summary4$slope<0 & summary4$p <0.05)),length(which(summary4$p<0.05)),0.5)$p.value**

**#### net diversification rate test per clade**

**length(which(tapply(summary4$net.t,as.character(summary4$clade),min)<0.06))/length(unique(summary4$clade))**

**binom.test(length(which(tapply(summary4$p.bar,as.character(summary4$clade),min)<0.06)),length(unique(summary4$clade)),0.5)$p.value**

**#### net diversification rate test per break**

**length(which(summary4$net.t<0.05))/(dim(summary4)[1])**

**binom.test(length(which(summary4$net.t<0.05)),(dim(summary4)[1]),0.5)$p.value**
